# Deep-red circularly polarised luminescent C_70_ derivatives

**DOI:** 10.1038/s41598-021-91451-5

**Published:** 2021-06-08

**Authors:** Haruka Kano, Hironobu Hayashi, Kyohei Matsuo, Michiya Fujiki, Hiroko Yamada, Naoki Aratani

**Affiliations:** 1 Division of Materials Science, Nara Institute of Science and Technology, 8916-5 Takayama-cho, Ikoma, Nara 630-0192 Japan; 2 Division of R&D, True2Materials PTE. Ltd., 8916-5 Takayama-cho, Ikoma, Nara 630-0192 Japan

**Keywords:** Near-infrared spectroscopy, Synthetic chemistry methodology, Circular dichroism

## Abstract

Optically active fullerenes, including C_60_ and C_70_ derivatives carrying organic substituents, are used in a range of applications because of their unique spectroscopic, catalytic, and chiral recognition properties. However, their inherent photoexcited chirality is yet to be elucidated because of their very poor fluorescence quantum yield (*Φ*_f_). We synthesised a new chiral C_70_ derivative, **X70A**, with 20% yield, by reacting bis-borylated xanthene with C_70_ in a one-step double addition reaction, followed by a successful optical resolution. The isolation of two separate **X70A** enantiomers was confirmed by mirror-image circular dichroism spectroscopy in the range of 300–750 nm. In toluene, the enantiomeric pair of **X70A** clearly revealed mirror-image circularly polarised luminescence (CPL) spectra with a high |*g*_lum_| value of 7.0 ×  10^−3^ at 690 nm. The first fullerene-based deep-red CPL of **X70A** should provide a new guideline for the design of chiral nanocarbon materials.

## Introduction

Achiral buckminsterfullerene (C_60_) and [5,6]-fullerene (C_70_) adopt highly symmetrical spherical and elliptical structures, respectively, allowing them to be utilised as *n*-type molecular semiconductors and building blocks of molecular conductors/magnets owing to the uniqueness of their energetically low-lying lowest unoccupied molecular orbitals (LUMOs)^1,2^. The LUMO characteristics of C_60_ and C_70_ in solution permit reversible acceptance and release of up to six electrons via an electrochemical redox process^3^. In addition, K_3_C_60_^4^ and Rb_3_C_60_^5^ have been shown to exhibit superconductivity at critical temperatures (*T*_c_) of ~ 20 and 30 K, respectively. In addition, a recent study indicated the possibility of *T*_c_ =  ~ 150 K when femtosecond laser pulses excite the phonon modes of K_3_C_60_ at 0.3 GPa^6^. Furthermore, the charge-transfer (CT) complex of C_60_ with an electron-donating molecule has been reported to undergo a paramagnetic-ferromagnetic transition at *T*_c_ =  ~ 17 K^7^. Moreover, photoinduced CT processes between fullerene derivatives and π-conjugated polymers have been found to efficiently generate electron and hole carriers with enhanced mobilities, thereby improving the performances of organic photovoltaic solar cells^8,9^.

Early photoluminescence (PL) studies have elucidated that fullerenes can emit fluorescence (FL), but in very low quantum yields (*Φ*_f_)^10–13^, and that the singlet (S_1_)–triplet (T_1_) intersystem crossing (ISC) occurs nearly quantitatively due to large spin–orbit coupling (SOC)^14^. Thus, a thermally activated delayed FL (TADF) is possible owing to the small S_1_–T_1_ energy gap (*ΔE*_S-T_)^15–19^. However, the lack of a high *Φ*_f_ at the S_1_–S_0_ transition remains an obstacle when fullerene derivatives are applied to several photonic applications.

Molecular chirality and helicity play key roles in biomolecular and human-made materials science, facilitating the introduction of a perturbation to the photoexcited and ground states. In solution, the majority of chiral organic luminophores exhibit circularly polarised luminescence (CPL) in the UV–visible region^20^. For example, a few helicenes, as helical nanocarbon molecules, have been shown to emit CPL in the visible region up to 800 nm^21,22^. However, nanocarbon materials that exhibit a large dissymmetry factor over long wavelength regions, such as the deep-red region, have rarely been investigated. This is due to the fact that the molecular design that can simultaneously achieve chirality and an effective π-conjugation whose absorption reaches the deep-red region is still unexplored. In this context, we attempt the rational design of the π surfaces of C_70_^23–25^ to produce chiral deep-red luminophores. As a result, a chiral C_70_ derivative, **X70A**, was synthesised by reacting bis-borylated xanthene with C_70_ in a one-step double addition reaction, followed by successful purification by high performance liquid chromatography (HPLC) using a chiral separation column.

Thus, we herein report the first deep-red mirror-image CPL spectra at 690 nm, originating from a pair of chiral fullerene derivatives, which are associated with the corresponding mirror-image circular dichroism (CD) spectra upon the dissolution of left-handed and right-handed **X70A** in toluene. We believe that the results obtained for this CPL-exhibiting **X70A** will provide useful guidelines for the future material design of nanocarbon light-emitting materials that emit in the deep-red to near infrared (NIR) region with high *g*-values^26–28^.

## Results

### Molecular and reaction design

**Figure 1:**
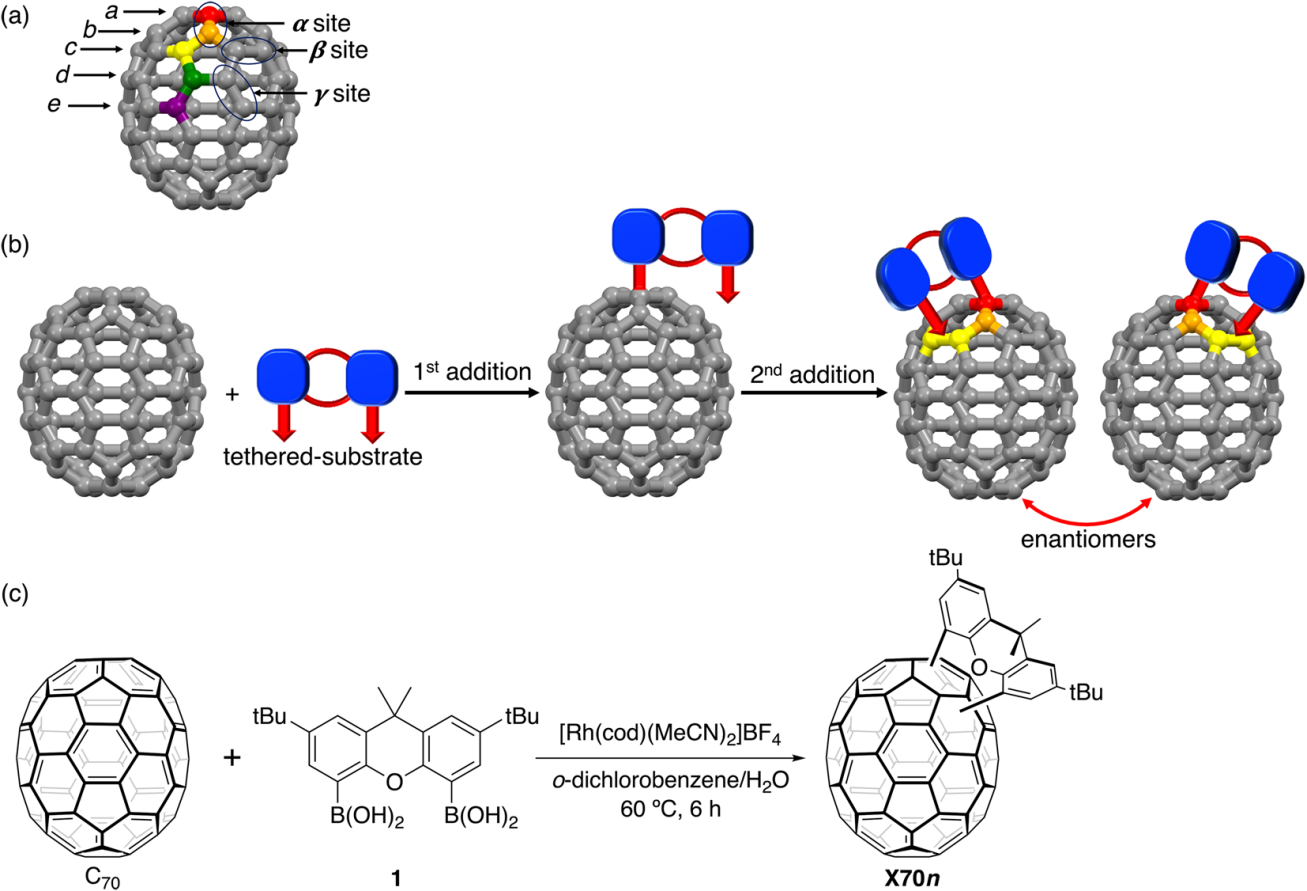
Schematic representation of the short distance tether-directed remote functionalisation of C_70_. (**a**) Ball-and-stick model of C_70_ with carbon labels: *a* (red), *b* (orange), *c* (yellow), *d* (green), and *e* (purple). (**b**) Sequential reaction of the tethered-substrate with C_70_, which is bound to carbon *a* in the first addition reaction. Here, the second addition reaction is limited to the *β* site. The reaction leads to chiral racemic products given the equivalence of the *β* sites on the two sides adjacent to the *α* site. (**c**) Reaction of C_70_ with bis-borylated xanthene **1** to afford **X70*n***. cod = cyclooctadiene.

Due to the fact that C_70_ possesses five non-equivalent carbon atoms (*a–e*, see Fig. 1a), the *a* and *b* atoms, which have a high angular distortion because of their proximities to the two poles, inherently exhibit a high reactivity toward several nucleophiles^29^. The reactivities of the *α*-site (*a*–*b* double bond) and the *β*-site (*c*–*c* double bond) are higher than those of other sites, facilitating the rational design of double bond-selective reactions^30–33^. It should be noted here that when two C–C double bonds are inequivalent to a symmetrical plane, C_60_ and C_70_ form chiral electronic structures due to symmetry breaking. In 1998, Diederich et al. synthesised racemic mixtures of bis-adducts of C_60_ and C_70_ by the Bingel reaction^34^, leading to the successful resolution of enantiomerically pure compounds.

Following a nucleophilic reaction of the substrate on the most reactive carbon *a* of C_70_, the second addition reaction can be performed regioselectively on the successive *β*-site (*c*–*c* double bond), which produces a chiral bis-adduct (Fig. 1b). This tether-directed remote functionalisation^35–37^ was first achieved for the selective preparation of a tris-adduct of C_60_^35^. In this study, we reacted two boronic acids, with a fixed short distance, on C_70_ with a rhodium catalyst using Itami’s method^38,39^. The xanthene skeleton was selected as a very short-tethered boronic acid. Bis-borylated xanthene **1** was prepared from 4,5-dibromo-2,7-di-*tert*-butyl-9,9-dimethylxanthene in 72% yield^40^.

### Isolation and characterisation of the X70 family

Bis-borylated xanthene **1** was reacted with C_70_ in the presence of a catalytic amount of [Rh(cod)(MeCN)_2_]BF_4_ in H_2_O/*o*-dichlorobenzene (1/4) at 60 °C for 6 h to yield the desired xanthene adducts (Fig. 1c)^39^. The obtained chromatogram (toluene/hexane = 1:1, *v*/*v*, COSMOSIL Buckyprep column, Nacalai Tesque Inc.) of the reaction mixture is shown in Supplementary Fig. S1. Mass spectrometric analysis of the products revealed that the first eluent contained bis-xanthene adducts, while the subsequent fractions contained the six mono-xanthene adducts (Supplementary Figs. S2–S7), i.e., **X70A**, **X70B**, **X70C**, **X70D**, **X70E**, and **X70F**. The obtained yield of **X70A** was moderately high (~ 20%).

**Figure 2:**
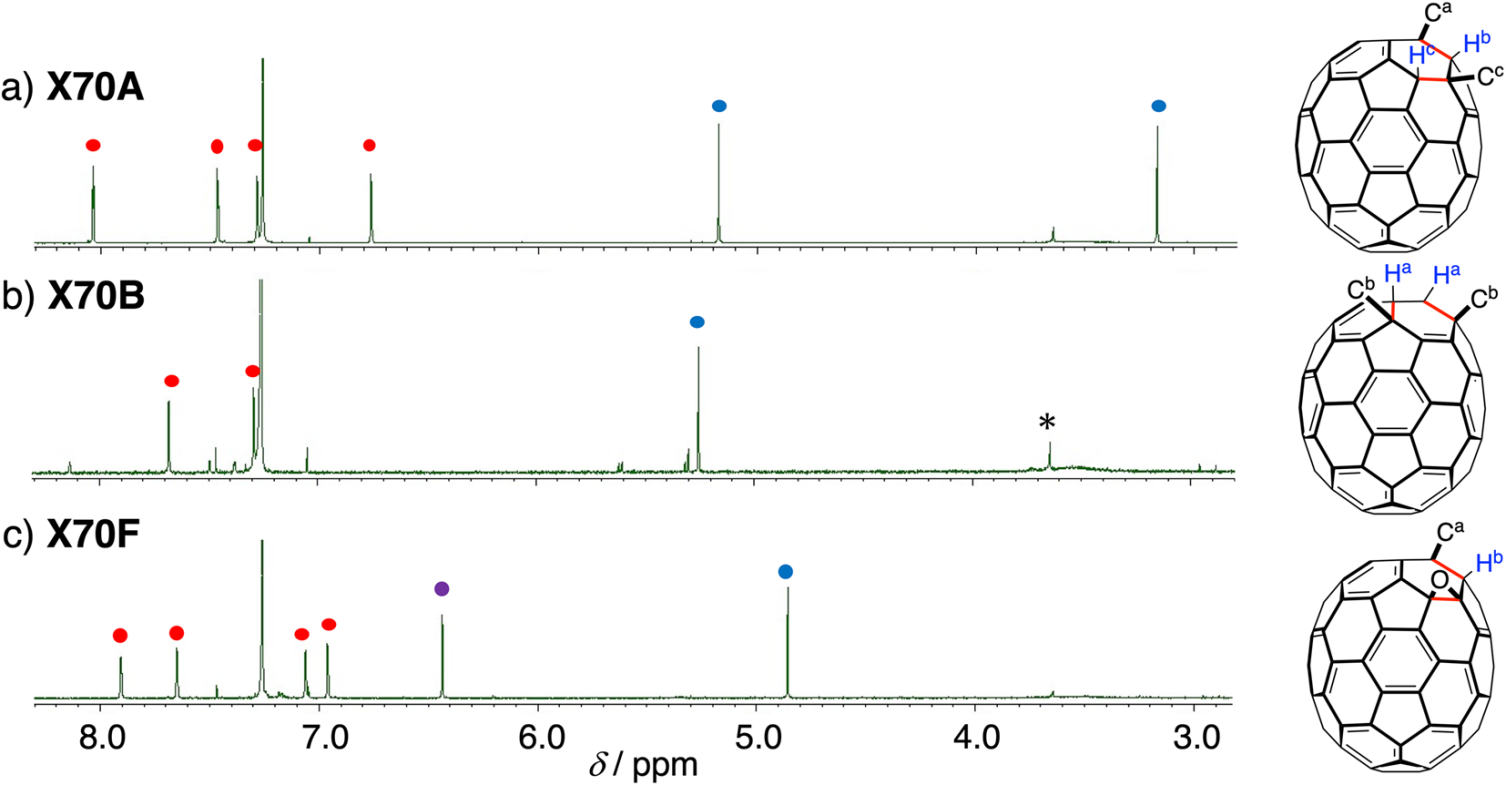
^1^H NMR spectra of a series of **X70*n*** and illustration of the replacement positions on C_70_. (**a**) **X70A** in CDCl_3_, (**b**) **X70B** in CDCl_3_, and (**c**) **X70F** in CDCl_3_. * indicates an impurity peak. Red circles are assigned to the peaks of xanthene, blue circles are assigned to the peaks of the fullerene protons, and a purple circle is assigned to the peak of the hydroxy group. C^a^, C^b^, and C^c^ represent the xanthene carbon atoms; H^a^, H^b^, and H^c^ represent the hydrogen atoms attached to carbons *a*, *b*, and *c*, respectively. The structures of **X70A**, **X70B**, and **X70F** determined by the single-crystal X-ray analysis are shown in Fig. 3.

The ^1^H and ^13^C nuclear magnetic resonance (NMR) spectra of **X70A**, **X70B**, and **X70F** are shown in Fig. 2 and Supplementary Figs. S8–S13. In Fig. 2, the red and blue circles indicate the proton peaks of xanthene and fullerene, respectively. Asymmetric **X70A** was characterised by four doublet peaks arising from the xanthene component, and two singlet peaks originating from the fullerene framework. The two singlet peaks observed for the fullerene framework indicate that the C_70_ carbon atoms at the 1,3-positions (carbons *a* and *c*) reacted preferentially. Formation of the highly symmetric **X70B** was confirmed by the observation of two doublet peaks corresponding to xanthene, and one singlet peak originating from the fullerene framework, indicating that the equivalent carbon *b* had reacted.

**Figure 3:**
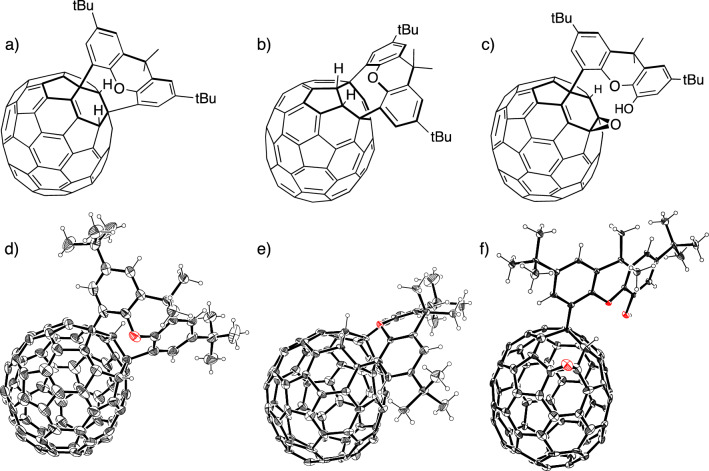
Structures of **X70A**, **X70B**, and **X70F**. Molecular structures of (**a**) **X70A**, (**b**) **X70B**, and (**c**) **X70F** and their ORTEP diagrams for single-crystal X-ray structures of (**d**) **X70A**, (**e**) **X70B**, and (**f**) **X70F** with 25% thermal ellipsoids. Solvent molecules and disordered parts are omitted for clarity.

**Figure 4:**
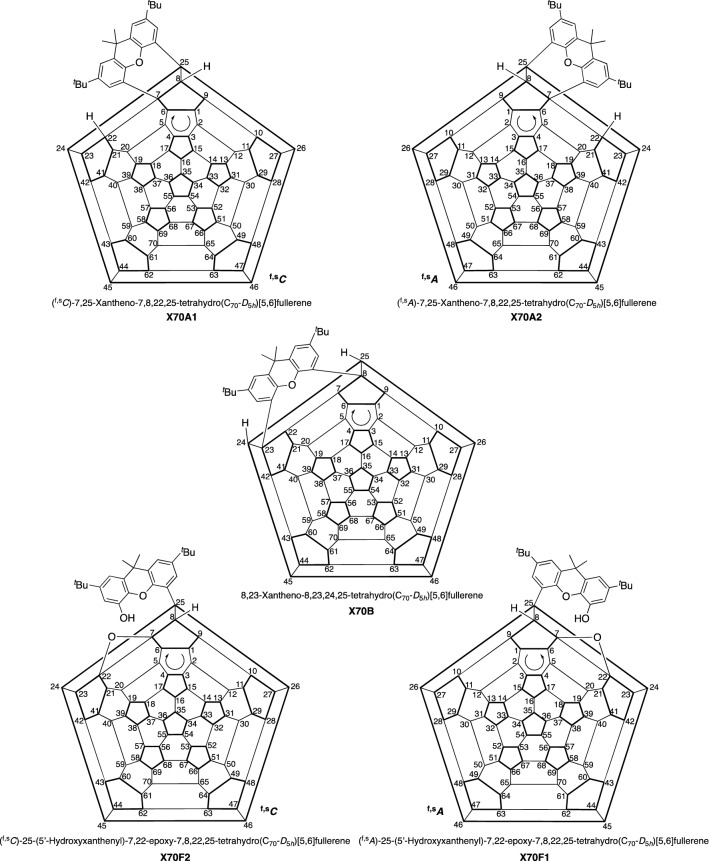
Schlegel diagrams of **X70A**, **X70B**, and **X70F** with enantiomeric numbering schemes: systematic numbering recommended by IUPAC; arrows indicate the direction of the numbering commencement. The full names for these compounds are listed in the Supplementary Information.

The structures of **X70A** and **X70B** were determined by single-crystal X-ray structure analysis (Fig. 3a,b,d,e, and Supplementary Tables S1–S2). **X70A** is a product of double addition reactions at the 1,3-positions of the top six-membered ring of C_70_, which results in the generation of a chiral structure. Alternatively, **X70B** was formed from the addition reaction at carbon *b* at the 1,4-positions of C_70_, as supported by the ^1^H NMR results. **X70A** and **X70B** were named using the official fullerene IUPAC nomenclature^25^, as indicated in Fig. 4 and Supplementary Fig. S14.

High-resolution matrix-assisted laser desorption/ionisation time-of-flight mass spectrometry (HR–MALDI–TOF–MS) of **X70E** and **X70F** detected **X70A** plus 16 and 32 mass units, respectively, suggesting that one and two oxygen atoms are inserted into the mono-xanthene adducts. The ^1^H NMR spectrum of **X70F** in CDCl_3_ was similar to that of **X70A**, exhibiting four doublet peaks originating from the xanthene component, in addition to two singlet peaks at 6.44 and 4.86 ppm (Fig. 2c).

The structure of **X70F** was unambiguously determined by X-ray diffraction analysis, where it was found that one of the C–C bonds was cleaved by oxygen (Figs. 3c,f, 4; Supplementary Table S3). This compound possesses one hydroxyl group on the xanthene moiety, and one oxygen atom at the *β*-site, indicating the presence of an epoxide structure. We confirmed that the singlet peak at 6.44 ppm in its ^1^H NMR spectrum disappeared after the addition of methanol-*d*_4_, indicating that this peak is derived from the hydroxyl group.

Although the formation mechanism of **X70F** remains unclear, we observed the conversion of **X70A** to **X70F** in solution under air, and so, **X70F** is considered to be produced by the air oxidation of **X70A** following its generation. The plausible structures and names for **X70C**, **X70D**, and **X70E** estimated from ^1^H and ^13^C NMR analyses (Supplementary Figs. S15–S19) are shown in Supplementary Fig. S20.

### Photophysical properties of the enantiomerically purified X70A and X70F

**Figure 5:**
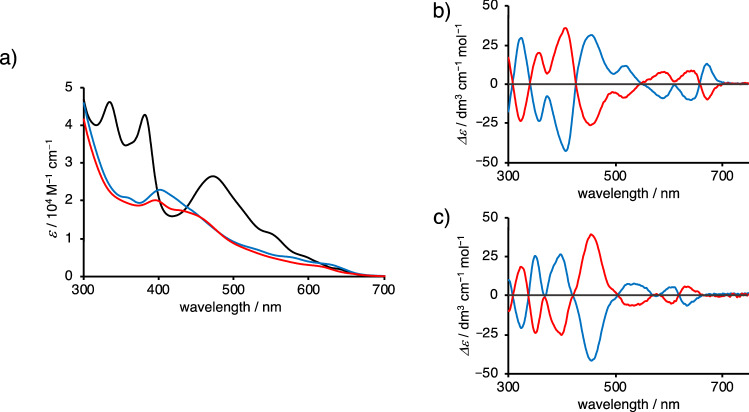
UV–visible absorption and CD spectra. (**a**) UV–vis absorption spectra of **X70A** (blue line), **X70F** (red line), and for comparison, C_70_ (black line) in toluene. (**b**) Blue and red lines represent the CD spectra in toluene for the first (**X70A1**: 9.6 × 10^−6^ M) and second (**X70A2**: 5.1 × 10^−6^ M) fractions, respectively. (**c**) Blue and red lines represent the CD spectra in toluene of the first (**X70F1**: 6.1 × 10^−6^ M) and second (**X70F2**: 5.1 × 10^−6^ M) fractions, respectively.

The UV–visible absorption spectra of **X70A**, **X70F**, and pristine C_70_ are shown in Fig. 5a and Supplementary Table S4. In the UV–visible absorption spectra of **X70A** and **X70F**, the characteristic peaks of C_70_ were greatly suppressed and broadened, exhibiting the typical absorption shape of monosubstituted fullerenes, as reported in the literature^30^.

Subsequently, the racemates of **X70A** and **X70F** were subjected to enantiomeric resolution using chiral separation column chromatography. Although the chromatogram of **X70A** in hexane/*i*-PrOH (4:1) did not show well-separated peaks after 24 cycles of the racemate, the first and second halves of the peaks clearly provided mirror-signed CD spectral profiles, indicating that enantiomeric resolution was possible (Supplementary Fig. S21). Furthermore, four repetitions of the enantiomeric resolution process using the first half of the peak succeeded in isolating mostly enantiomerically purified **X70A**^41,42^. Another chiral **X70A** was obtained using the second half of the peak of **X70A**, following six repetitions of the recycling enantiomeric resolution process (Supplementary Fig. S22). Enantiomerically purified **X70F**s were obtained using the same separation procedure (Supplementary Figs. S23, S24). As a result, **X70A1** and **X70A2**, as the first and second fractions, respectively, revealed ideal mirror-image CD spectra between 300 and 750 nm (Fig. 5b). The first and second fractions of **X70F** were named **X70F1** and **X70F2**, respectively (Fig. 5c).

To determine the absolute structures of chiral fullerenes **X70A** and **X70F**, we compared the experimental CD spectra with the density functional theory (DFT)-calculated spectra between 300 and 750 nm using the SpecDis software package (Supplementary Figs. S25–S27; Supplementary Tables S5–S7)^43,44^. Both CD spectra (i.e. for **X70A** and **X70F**) were simulated with high similarity factors (0.89 for **X70A** and 0.81 for **X70F**, respectively), and thus, the absolute structure of the second eluent **X70A2** was determined with high accuracy as (^f,s^*A*)-7,25-xantheno-7,8,22,25-tetrahydro(C_70_-*D*_5*h*_)[5,6]fullerene, and the first eluent **X70A1** was determined as (^f,s^*C*)-7,25-xantheno-7,8,22,25-tetrahydro(C_70_-*D*_5*h*_)[5,6]fullerene (Fig. 4). In addition, the absolute structure of the second eluent **X70F2** was determined as (^f,s^*C*)-25-(5′-hydroxyxanthenyl)-7,22-epoxy-7,8,22,25-tetrahydro(C_70_-*D*_5*h*_)[5,6]fullerene, while the first eluent **X70F1** was determined to be (^f,s^*A*)-25-(5′-hydroxyxanthenyl)-7,22-epoxy-7,8,22,25-tetrahydro(C_70_-*D*_5*h*_)[5,6]fullerene.

**Figure 6:**
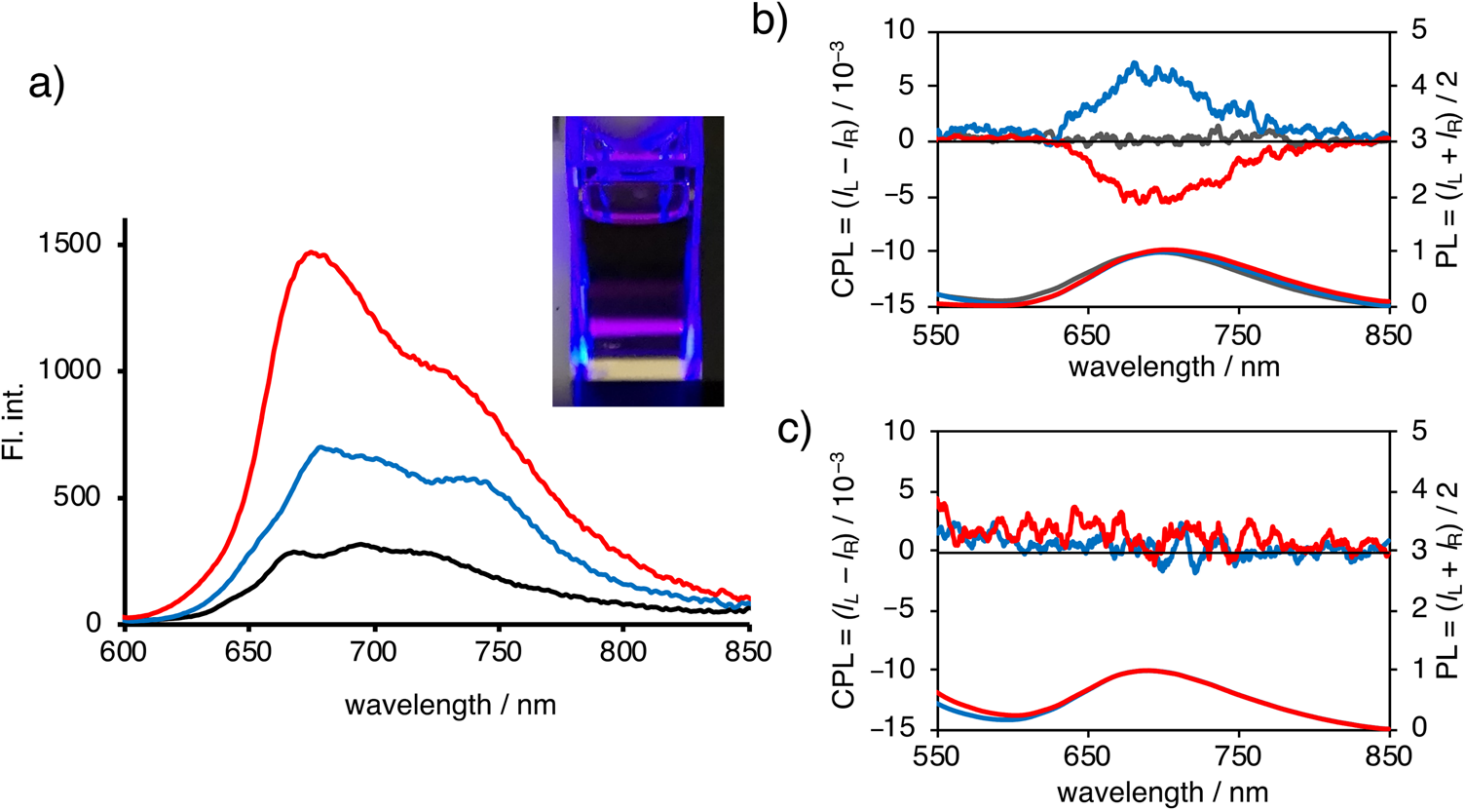
FL and CPL/PL spectra of **X70A** and **X70F** in toluene. (**a**) FL spectra of **X70A** (blue line), **X70F** (red line), and C_70_ (black line) in toluene excited at 500 nm with the absorbance adjusted at 0.1. Inset: photographic image of the deep-red FL from **X70A1** excited at 450 nm taken using a digital camera (Leica, ASA 6400, f1.8, SS1/4). (**b**) CPL (top) and PL (bottom) spectra of **X70A** in toluene excited at 410 nm. Blue, red, and grey lines represent the CPL/PL spectra of the first and second fractions of **X70A** and the intact C_70_, respectively. (**c**) CPL (top) and PL (bottom) spectra of **X70F** in toluene excited at 500 nm. Blue and red lines represent the CPL/PL spectra of the first and second fractions, respectively. Note that **X70F** showed no obvious CPL spectra.

The FL spectra of **X70A** and **X70F** are depicted in Fig. 6a along with that of C_70_ excited at 500 nm in degassed toluene at 20 °C for comparison. As indicated, **X70A** and **X70F** exhibit similar broad FL bands with vibronic shoulders in the range 600–850 nm. Based on a previous report that the *Φ*_f_ of C_70_ in toluene at 20 °C was ~ 0.06%^13^, the relative *Φ*_f_ values of **X70A** and **X70F** were determined to be 0.1 and 0.2%, respectively (Fig. 6a). These enhancements are probably due to a lowering symmetry accompanying a weak polarity led by two substituents of the fullerene π-systems; C_70_ adopts achiral *D*_5*h*_, while **X70A** and **X70F** are chiral *C*_1_-symmetry. Although the FL emission at ~ 700 nm cannot be detected with the naked eye, photosensitivity experiments carried out using a crystal silicon-based digital camera with detection up to 950 nm allowed the deep-red emission to be captured (inset of Fig. 6a). The FL lifetimes of **X70A** and **X70F** in deaerated toluene (3.0 × 10^−5^ M) were determined to be 0.99 and 1.31 ns, respectively, from which, we can determine the radiative (*k*_f_) and non-radiative (*k*_nr_) rate constants to be 1.0 × 10^6^ s^−1^ and 1.0 × 10^9^ s^−1^ for **X70A** and 1.5 × 10^6^ s^−1^ and 1.5 × 10^9^ s^−1^ for **X70F**, respectively.

**X70A1** and **X70A2** clearly display mirror-image CPL spectra (Fig. 6b). To the best of our knowledge, these CPL spectra are the first ones observed for fullerene-based compounds, although optically active C_76_^45^ and C_60_ adducts^46^ were previously found to exhibit FL. The absolute *g*_lum_ values of **X70A1** and **X70A2**, |*g*_lum_|, were moderately high: 7.0 × 10^−3^ (*λ*_ex_ = 410 nm, *λ*_em_ = 690 nm). It should be noted here that this *g*_lum_ value is among the highest in the deep-red to NIR regions for purely organic compounds that do not contain lanthanide metals^47^. No obvious CPL signals were observed for **X70F1** and **X70F2** (Fig. 6c). To account for this observation, we calculated the intersection of the electric and magnetic transition dipole moments of **X70A** and **X70F**. Interestingly, although **X70F** possesses two orthogonal electric and magnetic dipole moments, **X70A** does not (Supplementary Fig. S28). The orthogonal electric and magnetic dipole moments should result in the cancellation of the |*g*_lum_| values, thereby accounting for the reduced CPL observed for these compounds^48^. Since the π-conjugated systems of both **X70A** and **X70F** are identical, the reason for the difference in the angles between the two types of moments can be attributed to the subtle electronic and steric effects of the substituents. In recent years, molecular design to control the angle and strength of the two transition moments has been studied intensively^49,50^.

## Discussion

To date, investigations into the photochemistry of fullerenes have mainly focused on subsequent electron transfer after photoexcitation and triplet energy transfer, for example, through the generation of singlet oxygen. In this study, we successfully synthesised and characterised a family of xanthene-attached C_70_ derivatives, **X70*n***, via a facile one-step reaction from C_70_. Furthermore, enantiomeric separation from the racemates of **X70A** and **X70F** was also achieved, and these compounds were found to exhibit significantly strong emission properties than C_70_. The enantiomeric pair of **X70A** clearly revealed ideal mirror-image CPL spectra ranging from the deep-red to NIR regions with a high |*g*_lum_| value of 7 × 10^−3^ at 690 nm as a purely organic fluorophore. The corresponding *Φ*_f_ values are small because the singlet excited state of C_70_ is converted to the triplet excited state with an efficiency close to 100%. Also, the deep-red emission *Φ*_f_ of the fluorophores is small, owing to the smooth non-radiative pathway; hence, it is reasonable to aim for a molecular design that gives a large dissymmetry factor (*g*-value) for the deep-red luminophore.

It is well known that the transition electric dipole moment (***μ***) is larger than the transition magnetic dipole moment (***m***), and the *g*_lum_ value is inversely proportional to the absolute value of ***μ*** from the following relationship:

$$g_{{{\text{lum}}}} = {\text{4}}\left| \boldsymbol{m} \right|{\text{cos}}\theta /|\varvec{\mu }|$$

As can be seen from this equation, the molar absorption coefficient, which is directly proportional to the absolute value of ***μ***, and the *g*-value generally have a trade-off relationship^51^. Thus, fullerenes with smaller molar absorption coefficients should be used for the S_0_–S_1_ forbidden transitions to achieve chiral luminophores with high *g-*values.

Although the *Φ*_f_ value of **X70A** is small, this is the first step in developing chiral fullerene luminescence. In this study, by comparing C_70_ with **X70A** and **X70F**, we have found that the improvement of fluorescence quantum yield can be achieved by a lower symmetrization on the fullerene π-system associated with an introduction of polar substituent(s), and that the difference of substituted pattern on the fullerene also changes the strength and angle of electric and magnetic transition dipole moments (Supplementary Fig. S28) and thus greatly affects the dissymmetry factor. We believe that the strategy for developing a molecule that can exhibit a high *g*-value in the deep-red region is valuable and can be applied for molecular design in the near future^26–28^.

## Methods

### General methods

C_70_ (purchased from SES Research Inc.) was purified using a Buckyprep column and degassed at 20 °C prior to carrying out any spectroscopic measurements. See the Supplementary Methods for further details.

### Syntheses of X70A–X70F

A Schlenk flask was flame-dried under vacuum and filled with argon. Dry *o*-dichlorobenzene (140 mL) and H_2_O (36 mL) were added to this flask under a stream of argon. After performing three freeze–pump–thaw cycles, [Rh(cod)(MeCN)_2_]BF_4_ (45 mg, 0.18 mmol), C_70_ (500 mg, 0.59 mmol), and 2,7-di-*tert*-butyl-9,9-dimethylxanthene-4,5-diboronic acid (275 mg, 0.71 mmol) were added to the flask under a stream of argon. After stirring the mixture at 60 °C for 6 h, it was cooled to 20 °C. The organic layer was separated, passed through a pad of Celite and silica gel, and washed with toluene. The filtrate was concentrated and purified using a Buckyprep column (toluene/hexane (*v*/*v*) = 1:1 eluent) to afford **X70A** (140 mg, 20%), **X70B** (0.8 mg, 0.1%), **X70C** (12 mg, 1.0%), **X70D** (13 mg, 1.1%), **X70E** (0.7 mg, 0.1%), and **X70F** (3.1 mg, 0.3%) as brown solids. Spectral data for all compounds are provided in the Supplementary Information.

### HPLC purification

Preparative HPLC system was constructed using a *ϕ*10 × 250 mm Buckyprep column (Nacalai Tesque Inc., Kyoto, Japan), a JASCO UV-2075 Plus detector, and a JASCO PU-2086 Plus pump. Eluent: toluene/hexane = 1/1, *v*/*v* Temperature: 20 °C, flow rate: 3.0 mL/min, injection volume: 3.0 mL, and detection: UV absorption at 326 nm. Chiral resolutions of **X70A** and **X70F** were performed at 20 °C using a *ϕ*10 × 250 mm Cholester column (Nacalai Tesque Inc.) fitted to a recycling preparative HPLC system, which was constructed using a JASCO UV-2075 Plus detector and a JASCO PU-2086 Plus pump. Eluent: hexane/*i*-PrOH = 4/1 (*v*/*v*), flow rate: 4.5 mL/min, injection volume: 3.0 mL, and detection: UV absorption at 326 nm.

### CD measurements

The CD spectra were recorded using a JASCO J-820 spectropolarimeter.

### CPL measurements and analysis

Artefact-free PL and CPL spectra were obtained using a JASCO CPL-200 spectrofluoropolarimeter, which allowed us to avoid second- and third-order stray light due to diffraction grating. The spectrofluoropolarimeter was designed as a prism-based spectrometer with a forward scattering angle of 0°, and it was equipped with focusing and collecting lenses. In addition, a movable cuvette holder fitted on an optical rail enabled adjustment of the best focal point to maximise the PL and CPL signals. Simultaneous CPL and PL measurements allowed the quantitative evaluation of the degree of CPL efficiency relative to the PL, known as Kuhn’s dissymmetry factor (*g*_lum_), which is defined as *g*_lum_ = (*I*_L_ − *I*_R_)/[(*I*_L_ + *I*_R_)/2], where *I*_L_ and *I*_R_ refer to the intensities of the left- and right-handed CPL, respectively. The *g*_lum_ value was evaluated as *g*_lum_ = [ellipticity (mdeg)/32,980/ln10] / PL amplitude (Volts) at the CPL extremum.

## Supplementary Information

Supplementary Information 1.

## Data Availability

The authors declare that the data supporting the findings of this study are available within the paper and its supplementary information file. For full characterisation of new compounds and experimental details, see Supplementary Methods and Figures in the Supplementary Information file. The X-ray crystallographic coordinates for structures **X70A**, **X70B**, and **X70F** reported in this study have been deposited at the Cambridge Crystallographic Data Centre (CCDC) under deposition numbers 2013940–2013942. These data can be obtained free of charge from the CCDC via http://www.ccdc.cam.ac.uk/data_request/cif. All other data are available from the authors upon request.
