# Supplementary material for: Deep-red circularly polarised luminescent C70 derivatives
Source: Sci Rep. 2021 Jun 8;11:12072. doi: 10.1038/s41598-021-91451-5 (PMC8187662; doi:10.1038/s41598-021-91451-5)
Supplement: Supplementary file 1 — Supplementary Information 1. [file 41598_2021_91451_MOESM1_ESM.pdf]

Supporting Information
for

Deep-red circularly polarised luminescent C_{70} derivatives

Haruka Kano, Hironobu Hayashi, Kyohei Matsuo, Michiya Fujiki, Hiroko Yamada* and Naoki Aratani*

Instrumentation and Materials

^{1}H NMR (500 MHz and 600 MHz) and ^{13}C NMR (126 MHz and 150 MHz) spectra were recorded using a JEOL JNM-ECX500 spectrometer and a JEOL JNM-ECX600 spectrometer at ambient temperature by using tetramethylsilane as an internal standard. The high-resolution MS were measured by a JEOL JMS-700 MStation (MALDI-spiral TOF MS). X-ray crystallographic data were recorded at 90 K using a BRUKER-APEXII X-Ray diffractometer using Mo-Kα radiation equipped with a large area CCD detector.

UV/Vis absorption spectra were measured using a JASCO UV/Vis/NIR spectrophotometer V-570, and fluorescence spectra were measured using a JASCO PL spectrofluorometer FP-6600. Preparative high-performance liquid chromatography (HPLC) was performed using a ϕ10 × 250 mm Buckyprep (Nacalai Tesque), equipped with a JASCO UV-2075 Plus detector and a JASCO PU-2086 Plus pump. Chiral resolutions were performed at 20°C using a ϕ10 × 250 mm Cholester (Nacalai Tesque) fitted to a recycling preparative HPLC system (a JASCO UV-2075 Plus detector and a JASCO PU-2086 Plus pump). CD spectra were recorded using a JASCO J-820 spectropolarimeter. CPL and PL spectra were collected using a JASCO CPL-200 spectrofluoropolarimeter.

TLC and gravity column chromatography were performed on Art. 5554 (Merck KGaA) silica gel plates and silica gel 60N (Kanto Chemical), respectively. All solvents and chemicals were reagent-grade quality, obtained commercially, and used without further purification. For spectral measurements, spectral-grade toluene was purchased from Nacalai Tesque. Xanthene **1** was prepared according to literature.[S1]

Experimental Section

Xanthene-attached C_{70} X70*n*:[S2] A Schlenk flask was flame-dried under vacuum and filled with argon. To this flask were added dry *o*-dichlorobenzene (140 mL) and H_2O (36 mL) under a stream of argon. After performing three freeze-pump-thaw cycles, $[Rh(cod)(MeCN)_2]BF_4$ (45 mg, 0.18 mmol), C_{70} (500 mg, 0.59 mmol), and 2,7-di-*tert*-butyl-9,9-dimethylxanthene-4,5-diboronic acid (275 mg, 0.71 mmol) were added to the flask under a stream of argon. After stirring the mixture at 60°C for 6 h, the mixture was cooled to room temperature. The organic layer was separated and passed through a pad of Celite and silica gel with washing with toluene. The filtrate was concentrated and purified by Buckyprep column (toluene/hexane (*v*/*v*) = 1:1 eluent) to afford **X70A** (140 mg, 20%), **X70B** (0.8 mg, 0.1%), **X70C** (13 mg, 1.0%), **X70D** (13 mg, 1.1%), **X70E** (0.7 mg, 0.1%) and **X70F** (3.1 mg, 0.3%) as brown solids. **X70A:** ^{1}H NMR (500 MHz, $CDCl_3$): δ = 8.03 (d, J = 2.0 Hz, 1H), 7.46 (d, J = 1.5 Hz, 1H), 7.28 (d, J = 1.5 Hz, 1H), 6.76 (d, J = 2.0 Hz, 1H), 5.17 (s, 1H), 3.17 (s, 1H), 1.91 (s, 3H), 1.89 (s, 3H), 1.52 (s, 9H) and 1.20 (s, 9H) ppm; ^{13}C NMR (150 MHz, $CDCl_3$): δ = 159.48, 155.70, 154.92, 152.98, 152.71, 152.68, 152.34, 152.00, 151.15, 151.13, 150.34, 149.93, 149.89, 149.78, 149.78, 149.65, 149.02, 148.97, 148.70, 148.64, 148.26, 148.17, 147.99, 147.62, 147.31, 147.29, 147.12, 147.09, 147.01, 146.98, 146.86, 146.76, 146.65, 146.41, 146.33, 146.10, 145.98, 145.82, 145.69, 145.64, 145.44, 145.36, 145.29, 145.05, 144.95, 144.92, 144.54, 144.21, 144.17, 144.04, 143.53, 143.36, 143.06, 142.79, 141.99, 141.86, 140.15, 139.90, 139.71, 139.24, 136.15, 135.71, 135.50, 135.18, 133.88, 133.79, 132.85, 132.79, 132.59, 132.23, 131.66, 131.46, 131.10, 128.90, 121.29, 120.63, 119.79, 118.94, 68.01, 63.19, 58.15, 50.41, 41.02, 35.63, 35.24, 32.22, 31.89, 29.02 and 22.70 ppm (89 signals out of 89 expected); HR-MS (Spiral MALDI): *m/z*: calcd for $C_{93}H_{30}O$, 1162.2291 $[M]^+$; found: 1162.2288; UV-vis (toluene): λ_{max} (ε [10^3 M^{-1} cm^{-1}]) = 361 (20.3), 401 (22.1), 533 (7.15), 579 (4.93) and 632 (2.92) nm. **X70B**: ^{1}H NMR (500 MHz, $CDCl_3$): δ = 7.68 (d, J = 2.0 Hz, 2H), 7.29 (d, J = 1.5 Hz, 2H), 5.26 (s, 2H), 1.79 (s, 3H), 1.64 (s, 3H) and 1.28 (s, 18H) ppm; ^{13}C NMR (150 MHz, $CDCl_3$): δ = 156.45, 154.39, 153.63, 153.38, 151.95, 151.26, 151.17, 150.57, 150.38, 150.30, 150.24, 149.62, 149.48, 149.34, 149.02, 148.80, 148.31, 148.02, 147.51, 147.46, 147.33, 147.29, 146.35, 145.48, 144.71, 143.36, 143.12, 141.31, 140.81, 140.81, 136.38, 135.13, 134.79, 134.57, 133.73, 133.67, 132.67, 132.23, 132.18, 120.85, 120.35, 59.99, 50.69, 39.48, 35.37, 32.12, 31.92 and 29.71 ppm (48 signals out of 48 expected); HR-MS (Spiral MALDI): *m/z*: calcd for $C_{93}H_{30}O$, 1162.2291 $[M]^+$; found: 1162.2304; UV-vis (toluene): λ_{max} (relative intensity) = 345 (1.00), 375 (0.85), 395 (0.83), 436 (0.94), 472 (0.76), 540 (0.46), 637 (0.12) and 688 (0.10) nm. **X70C**: ^{1}H NMR (500 MHz, $CDCl_3$): δ = 8.14 (d, J = 1.5 Hz, 1H), 7.50 (d, J = 1.5 Hz, 1H), 7.38 (d, J = 2.0 Hz, 1H), 7.27 (d, J = 2.0 Hz, 1H), 5.61 (d, J = 7.0 Hz, 1H), 5.31 (d, J = 7.0 Hz, 1H), 1.94 (s, 3H), 1.69 (s, 3H), 1.53 (s, 9H) and 1.27 (s, 9H) ppm; ^{13}C NMR: N.D.; HR-MS (Spiral MALDI): *m/z*: calcd for $C_{93}H_{30}O$, 1162.2291 $[M]^+$; found: 1162.2292. **X70D**: ^{1}H NMR (500 MHz, CD_2Cl_2): δ = 8.18 (d, J = 2.5 Hz, 1H), 7.69 (d, J = 2.5 Hz, 1H), 7.39 (d, J = 8.5 Hz, 1H), 7.24 (dd, J = 8.5, 2.5 Hz, 1H), 4.83 (s, 1H), 1.81 (s, 6H), 1.34 (s, 9H), and 1.26 (s, 9H) ppm; ^{13}C NMR: (150 MHz, $CDCl_3$): δ = 166.29, 159.57, 155.58, 151.61, 151.51, 151.10, 151.05, 150.30, 149.99, 149.84, 149.62, 149.42, 149.22, 149.20, 148.06, 148.02, 147.57, 147.51, 147.11, 146.77, 146.57, 146.52, 146.32, 146.28, 146.11, 145.80, 144.91, 143.46, 143.28, 143.21, 142.88, 141.56, 141.41, 141.41, 140.65, 134.33, 134.24, 134.19, 131.80, 131.53, 131.34, 131.10, 129.41, 124.78, 124.48, 123.57, 122.95,

115.99, 59.14, 54.83, 35.22, 34.95, 34.71, 32.90, 31.94 and 31.69 ppm (56 signals out of 56 expected); HR-MS (Spiral MALDI): *m/z*: calcd for $C_{93}H_{30}O$, 1162.2291 $[M]^+$; found: 1162.2308; UV-vis (toluene): λ_{max} (relative intensity) = 341 (1.00), 398 (0.86), 468 (0.61), 536 (0.33) and 662 (0.06) nm. **X70E**: ^{1}H NMR (500 MHz, $CDCl_3$): 8.27 (d, J = 2.0 Hz, 1H), 7.67 (d, J = 2.0 Hz, 1H), 7.29 (d, J = 2.0 Hz, 1H), 7.20 (d, J = 2.0 Hz, 1H), 4.95 (s, 1H), 4.81 (s, 1H), 1.98 (s, 3H), 1.58 (s, 3H), 1.57 (s, 9H), and 1.32 (s, 9H) ppm; ^{13}C NMR: (150 MHz, $CDCl_3$): δ = 159.20, 154.95, 154.94, 151.59, 151.56, 151.56, 151.12, 150.99, 150.29, 150.04, 149.79, 149.79, 149.34, 149.20, 149.18, 147.73, 147.55, 147.37, 147.30, 147.08, 146.59, 146.32, 146.26, 146.21, 145.26, 143.69, 143.48, 143.30, 143.27, 142.99, 140.32, 140.18, 137.40, 136.08, 134.31, 134.25, 133.62, 131.83, 131.69, 131.55, 131.35, 130.08, 126.19, 124.23, 120.15, 113.95, 111.53, 59.19, 54.53, 35.14, 35.08, 34.72, 33.27, 31.82, 31.56 and 29.86 ppm (56 signals out of 56 expected); HR-MS (Spiral MALDI): *m/z*: calcd for $C_{93}H_{29}O_2$, 1177.2162 $[M - H]^-$; found: 1177.2173; UV-vis (toluene): λ_{max} (relative intensity) = 358 (1.00), 398 (0.87), 588 (0.18) and 657 (0.07) nm. **X70F**: ^{1}H NMR (500 MHz, $CDCl_3$): δ = 7.90 (d, J = 2.5 Hz, 1H), 7.65 (d, J = 2.5 Hz, 1H), 7.06 (d, J = 2.5 Hz, 1H), 6.96 (d, J = 2.5 Hz, 1H), 6.44 (s, 1H), 4.86 (s, 1H), 1.83 (s, 6H), 1.49 (s, 9H), and 1.34 (s, 9H) ppm; ^{13}C NMR (150 MHz, $CDCl_3$): δ = 169.03, 168.87, 155.25, 152.36, 152.14, 151.72, 151.33, 151.15, 150.96, 150.77, 150.67, 150.64, 150.56, 150.45, 150.14, 150.01, 149.71, 149.68, 149.57, 149.23, 149.13, 149.01, 148.82, 148.65, 148.55, 148.27, 148.09, 148.06, 147.97, 147.73, 147.58, 147.55, 147.51, 147.28, 147.01, 146.68, 146.66, 146.43, 146.41, 146.33, 146.01, 145.88, 145.82, 145.65, 145.13, 144.73, 144.13, 144.07, 144.01, 143.88, 143.73, 143.64, 143.56, 142.03, 141.33, 139.17, 138.13, 137.79, 136.17, 135.84, 135.14, 134.20, 133.99, 133.48, 133.03, 132.53, 132.38, 132.22, 131.76, 131.60, 131.53, 131.44, 131.40, 130.07, 126.37, 124.12, 113.98, 111.51, 81.16, 70.81, 59.58, 48.98, 35.13, 34.75, 33.39, 33.10, 31.79, 31.58 and 29.86 (89 signals out of 89 expected); HR-MS (Spiral MALDI): *m/z*: calcd for $C_{93}H_{30}O_3Na$, 1217.2087 $[M]^+$; found: 1217.2089; UV-vis (toluene): λ_{max} (ε [10^3 M^{-1} cm^{-1}]) = 399 (20.0), 455 (15.9) and 623 (2.32) nm.

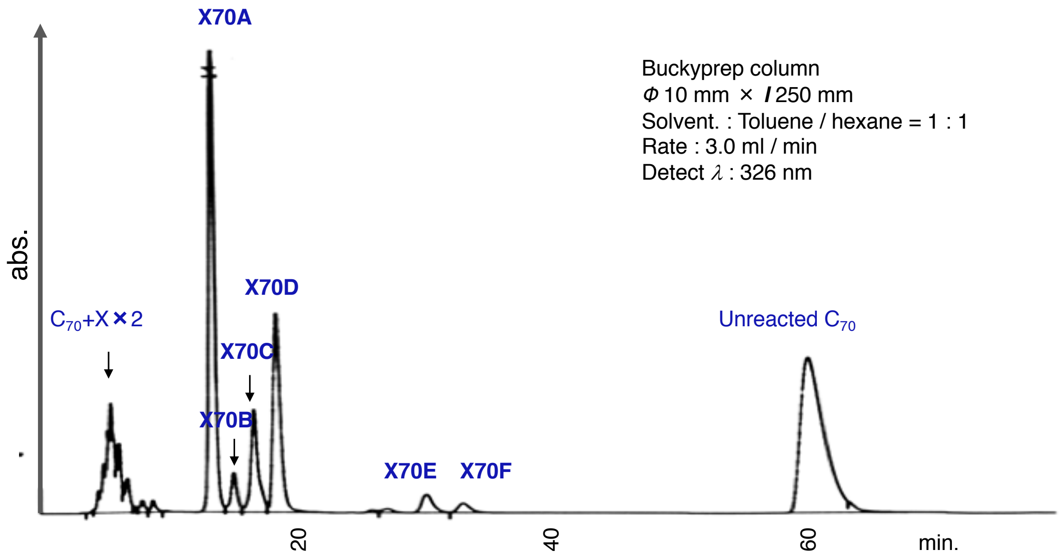

Supplementary Fig. 1. Buckyprep HPLC chromatogram of the reaction of C_{70} with **1**.

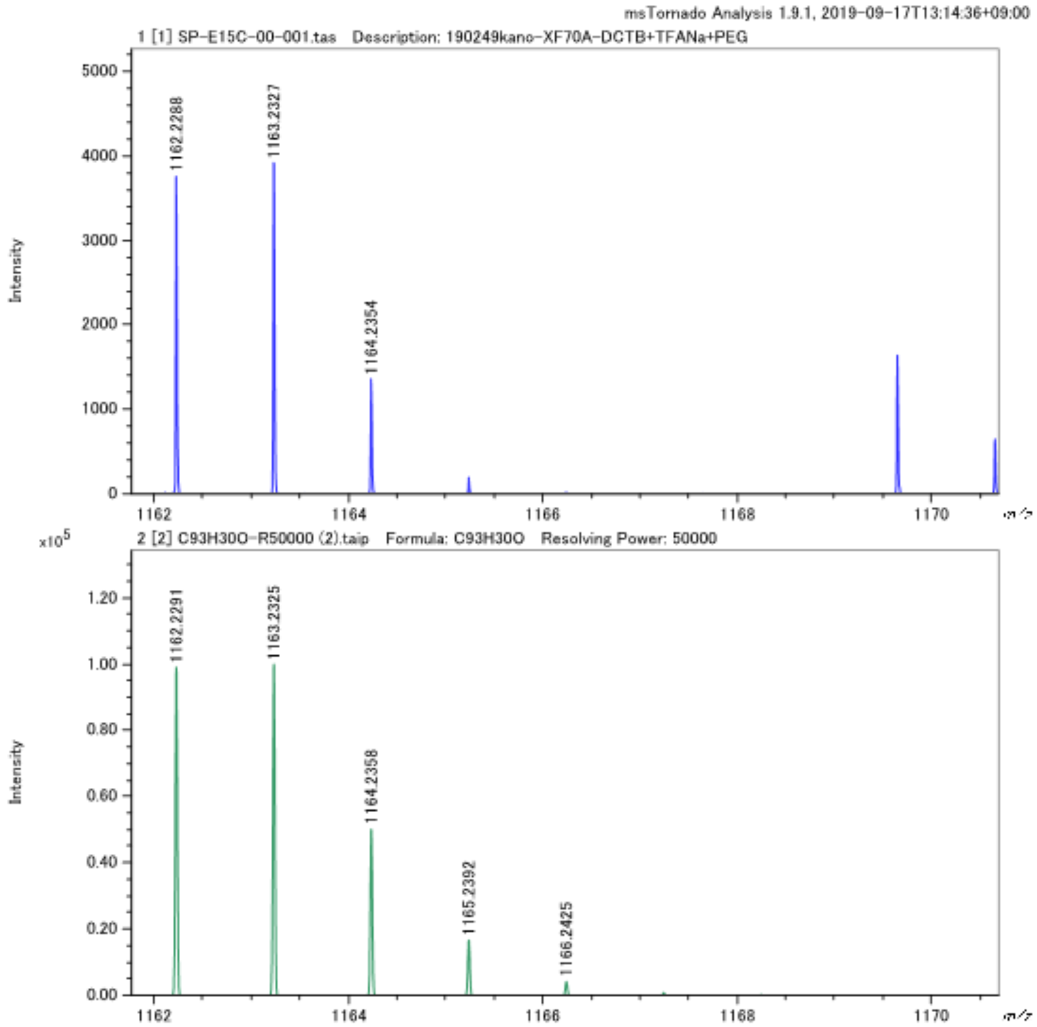

Elemental Composition Estimation

Parameters:

Mass	Tolerance	Electron Mode	Charge	DBE Range	Max Results
1162.22884 ± 0.00581	5.0 ppm	Odd/Even	+1	−0.5 − 200.0	100

Elements

C 0 − 100 H 0 − 200 N 0 − 10 O 0 − 10 Na 0 − 0

Results:

#	Formula	Mass	DBE	Abs. Error (u)	Error (u)	Error (ppm)
1	C80 H157 N2 O	1162.22904	3.5	0.00020	−0.00020	−0.17
2	C93 H30 O	1162.22912	79.0	0.00028	−0.00028	−0.24
3	C77 H30 N8 O6	1162.22828	67.0	0.00056	0.00056	0.48
4	C79 H32 N5 O7	1162.22962	66.5	0.00078	−0.00078	−0.67
5	C91 H28 N3	1162.22777	79.5	0.00107	0.00107	0.92
6	C78 H155 N5	1162.22770	4.0	0.00114	0.00114	0.98
7	C76 H34 N4 O10	1162.22694	62.0	0.00190	0.00190	1.63
8	C80 H28 N9 O3	1162.23096	71.5	0.00212	−0.00212	−1.83
9	C81 H34 N2 O8	1162.23097	66.0	0.00213	−0.00213	−1.83
10	C74 H32 N7 O9	1162.22560	62.5	0.00324	0.00324	2.79
11	C82 H30 N6 O4	1162.23230	71.0	0.00346	−0.00346	−2.98
12	C88 H30 N2 O3	1162.22509	75.0	0.00375	0.00375	3.22
13	C75 H157 N4 O3	1162.22502	−0.5	0.00382	0.00382	3.29

Supplementary Fig. 2. HR-Spiral-MALDI-TOF mass spectrum of **X70A**.

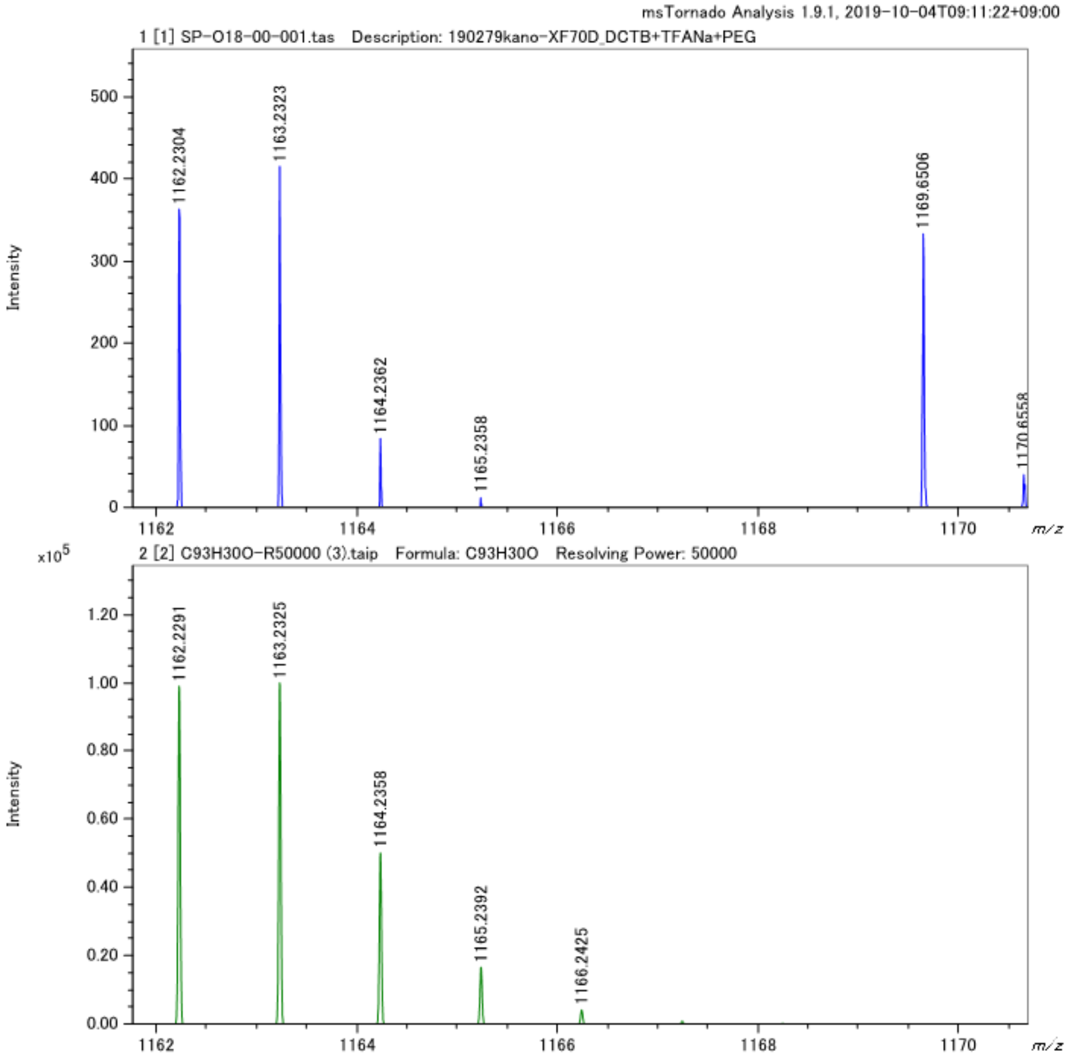

Elemental Composition Estimation

Parameters:

Mass	Tolerance	Electron Mode	Charge	DBE Range	Max Results
1162.23037 ± 0.00581	5.0 ppm	Odd/Even	+1	−0.5 − 200.0	100

Elements

C 90 − 100 H 20 − 40 O 0 − 1

Results:

#	Formula	Mass	DBE	Abs. Error (u)	Error (u)	Error (ppm)
1	C93 H30 O	1162.22912	79.0	0.00125	0.00125	1.08

Supplementary Fig. 3. HR-Spiral-MALDI-TOF mass spectrum of **X70B**.

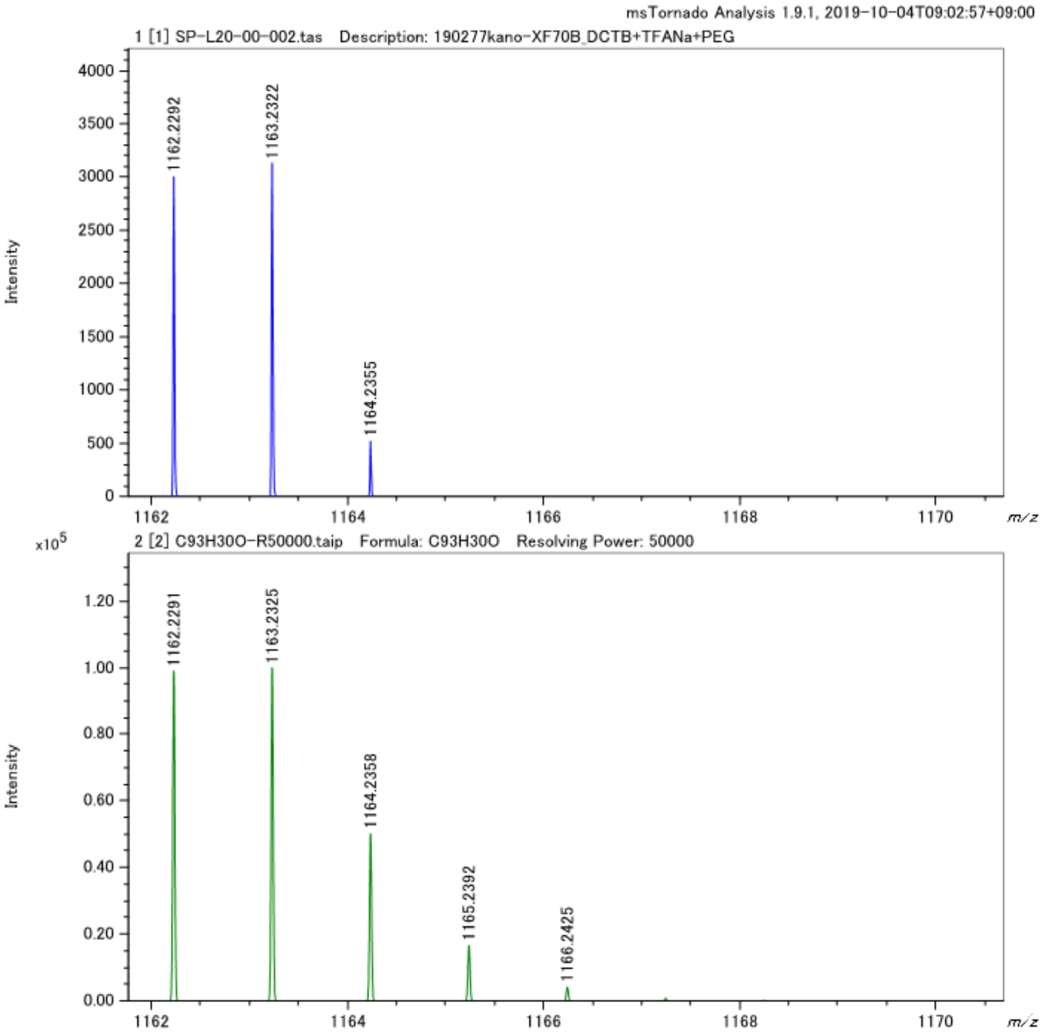

Elemental Composition Estimation

Parameters:

Mass	Tolerance	Electron Mode	Charge	DBE Range	Max Results
1162.22923 ± 0.00581	5.0 ppm	Odd/Even	+1	−0.5 − 200.0	100

Elements

C 90 − 100 H 20 − 40 O 0 − 1

Results:

#	Formula	Mass	DBE	Abs. Error (u)	Error (u)	Error (ppm)
1	C93 H30 O	1162.22912	79.0	0.00011	0.00011	0.10

Supplementary Fig. 4. HR-Spiral-MALDI-TOF mass spectrum of **X70C**.

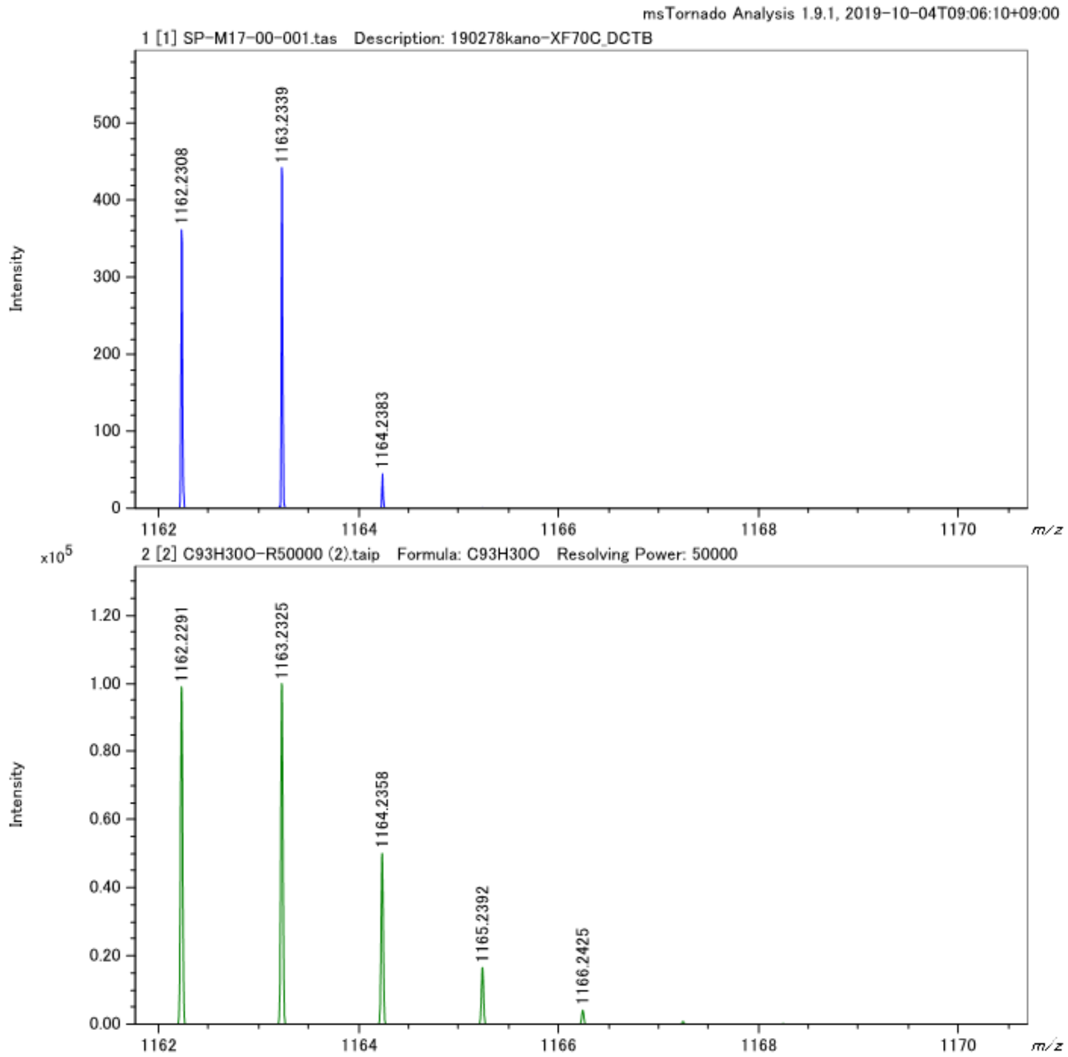

Elemental Composition Estimation

Parameters:

Mass	Tolerance	Electron Mode	Charge	DBE Range	Max Results
1162.23075 ± 0.00581	5.0 ppm	Odd/Even	+1	−0.5 − 200.0	100

Elements

C 90 − 100 H 20 − 40 O 0 − 1

Results:

#	Formula	Mass	DBE	Abs. Error (u)	Error (u)	Error (ppm)
1	C93 H30 O	1162.22912	79.0	0.00164	0.00164	1.41

Supplementary Fig. 5. HR-Spiral-MALDI-TOF mass spectrum of **X70D**.

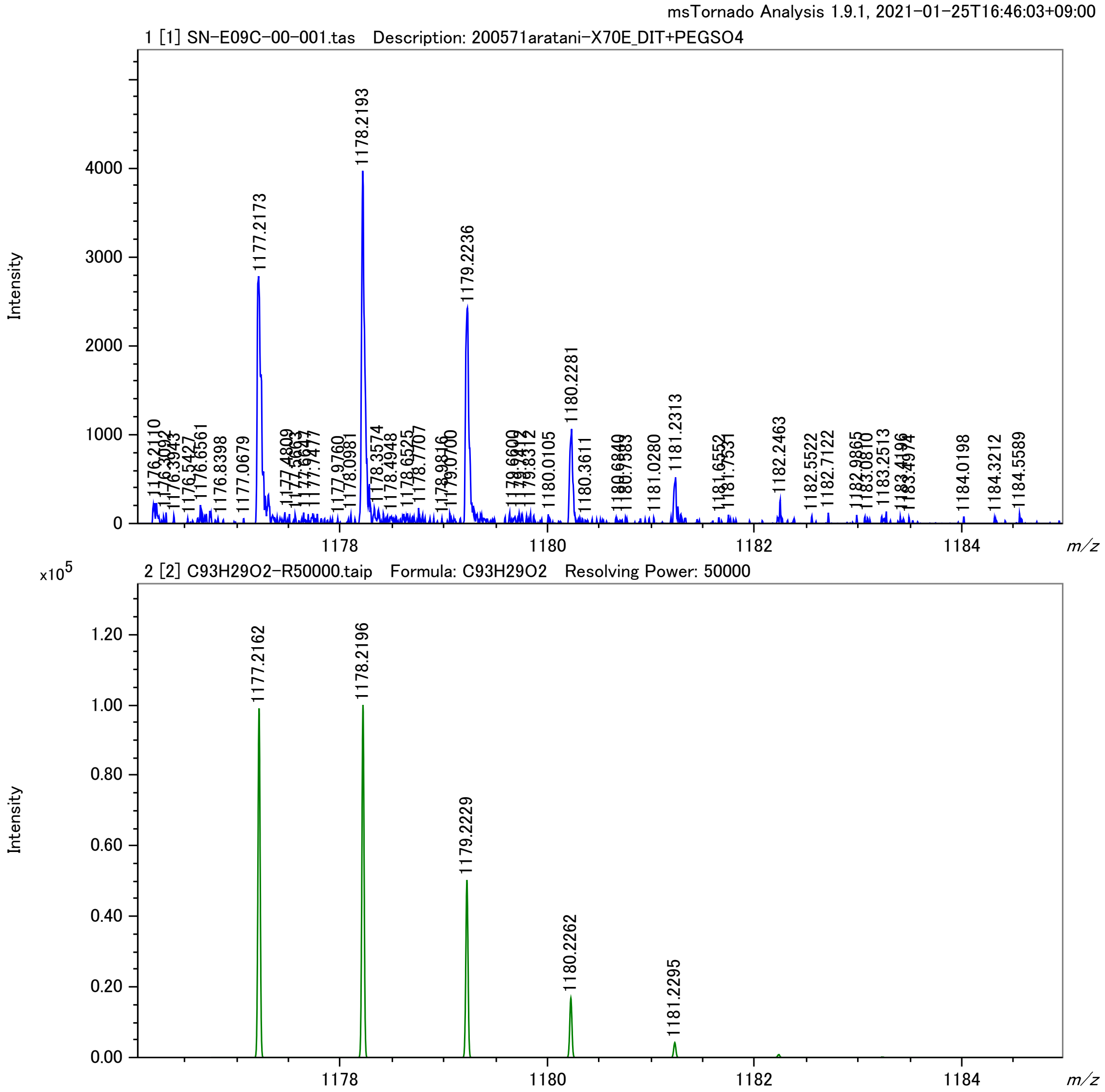

Elemental Composition Estimation

Parameters:

Mass	Tolerance	Electron Mode	Charge	DBE Range	Max Results
1177.21726 ± 0.00589	5.0 ppm	Odd/Even	+1	−0.5 − 200.0	100

Elements

C 0 − 100 H 0 − 40 O 0 − 5 Na 0 − 1

Results:

#	Formula	Mass	DBE	Abs. Error (u)	Error (u)	Error (ppm)
1	C93 H29 O2	1177.21621	79.5	0.00105	0.00105	0.90
2	C91 H30 O2 Na	1177.21380	76.5	0.00346	0.00346	2.94

Supplementary Fig. 6. HR-Spiral-MALDI-TOF mass spectrum of **X70E**.

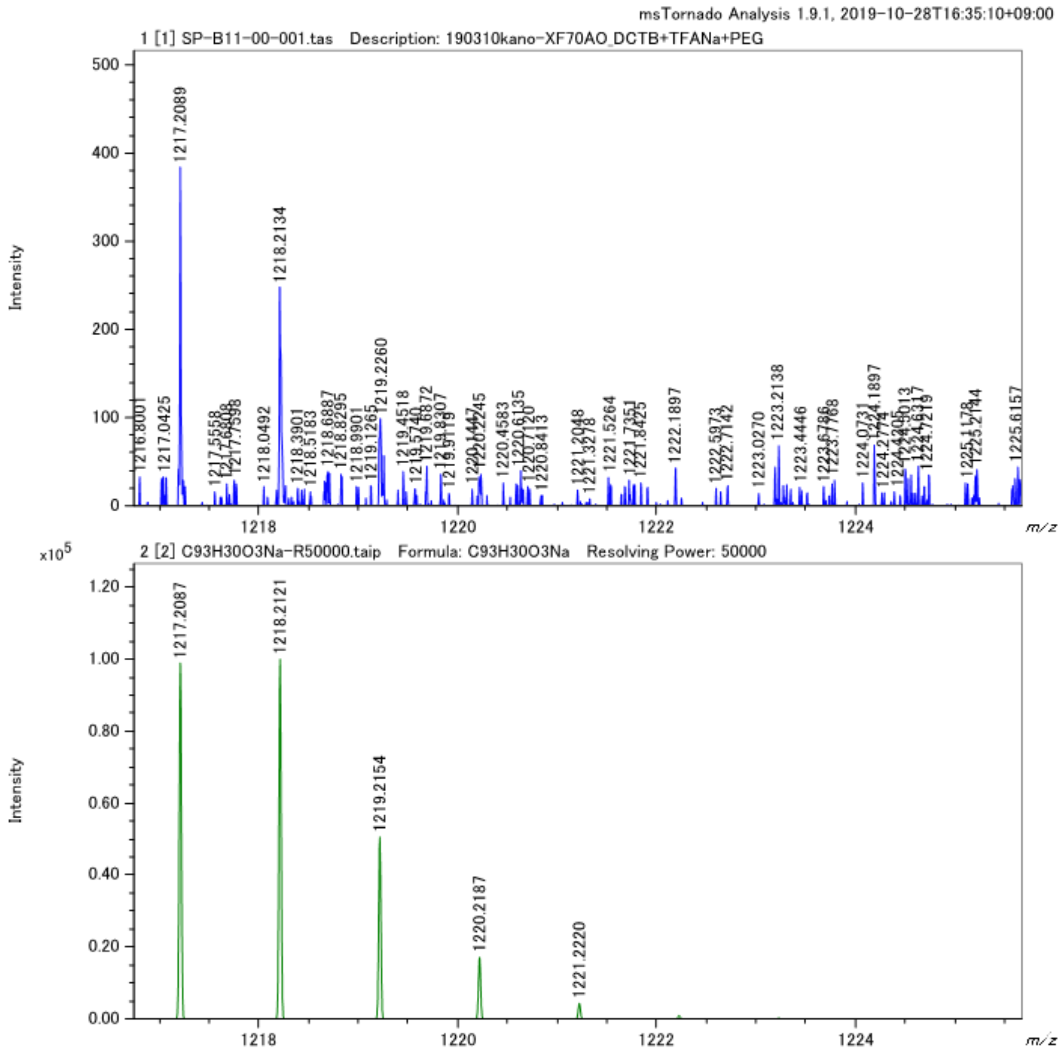

Elemental Composition Estimation

Parameters:

Mass	Tolerance	Electron Mode	Charge	DBE Range	Max Results
1217.20893 ± 0.00609	5.0 ppm	Odd/Even	+1	−0.5 − 200.0	100

Elements

C 0 − 100 H 0 − 40 O 0 − 3 Na 0 − 1

Results:

#	Formula	Mass	DBE	Abs. Error (u)	Error (u)	Error (ppm)
1	C93 H30 O3 Na	1217.20872	78.5	0.00022	0.00022	0.18
2	C95 H29 O3	1217.21112	81.5	0.00219	−0.00219	−1.80

Supplementary Fig. 7. HR-Spiral-MALDI-TOF mass spectrum of **X70F**.

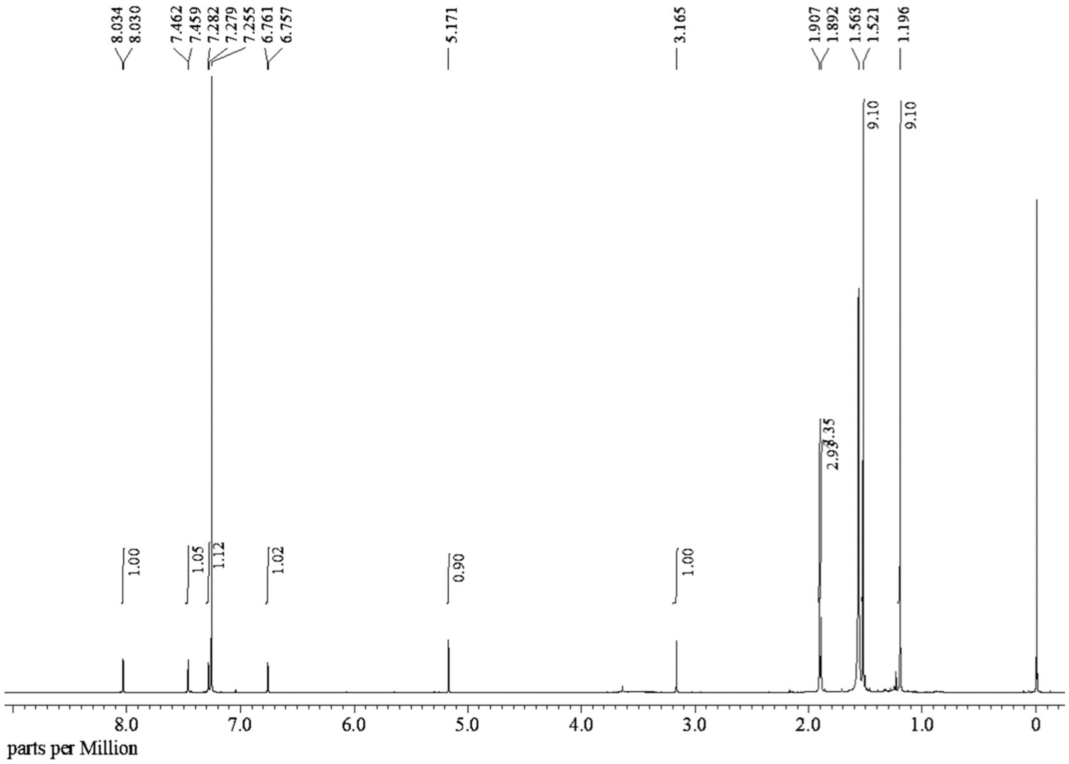

Supplementary Fig. 8. ^{1}H NMR spectrum of **X70A** in $CDCl_3$ at room temperature.

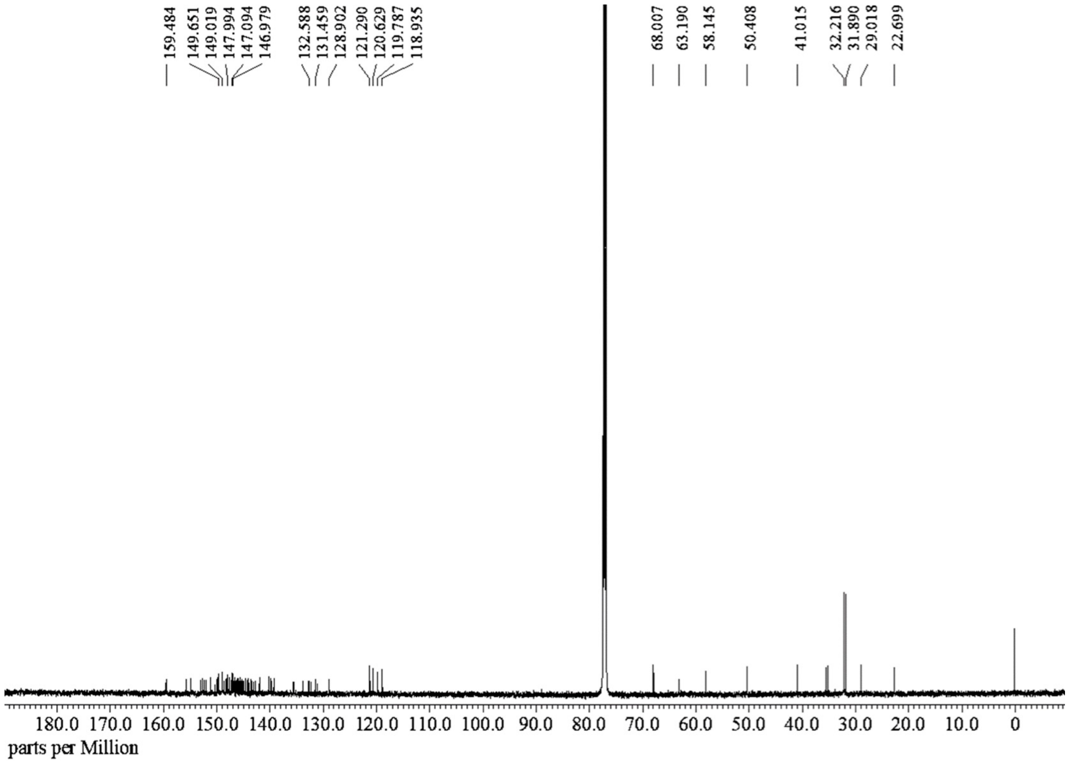

Supplementary Fig. 9. ^{13}C NMR spectrum of **X70A** in $CDCl_3$ at room temperature.

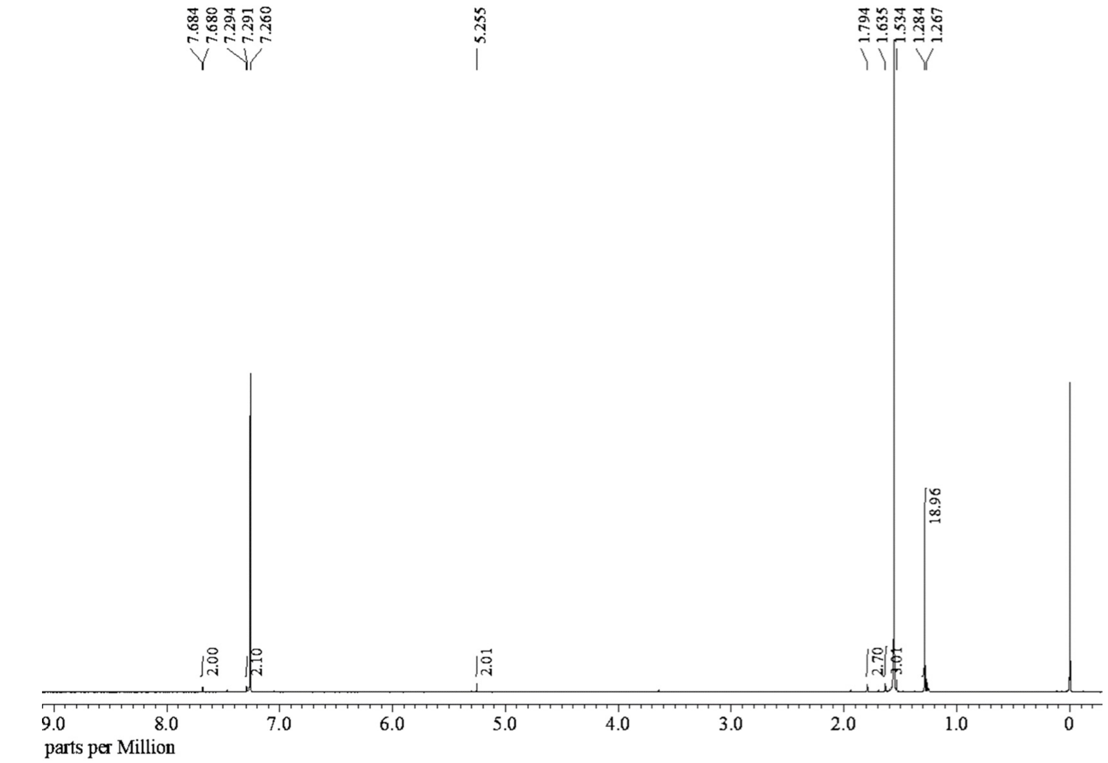

Supplementary Fig. 10. ^{1}H NMR spectrum of **X70B** in $CDCl_3$ at room temperature.

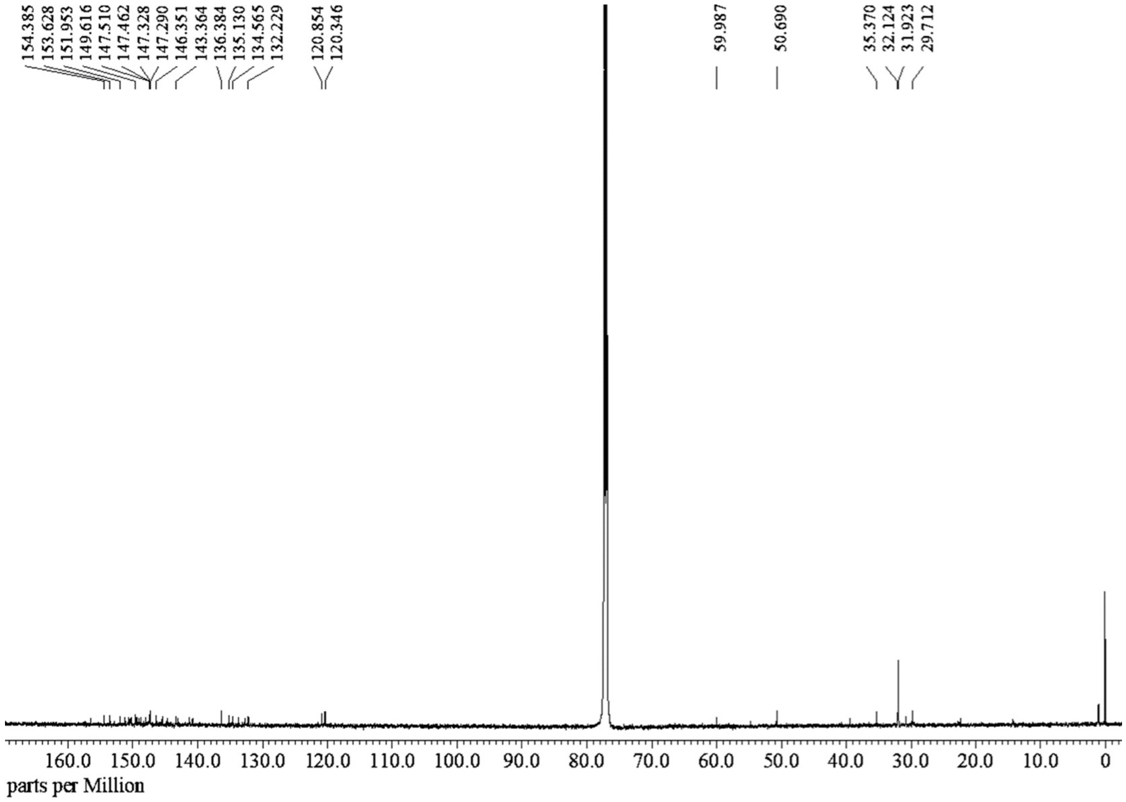

Supplementary Fig. 11. ^{13}C NMR spectrum of **X70B** in $CDCl_3$ at room temperature.

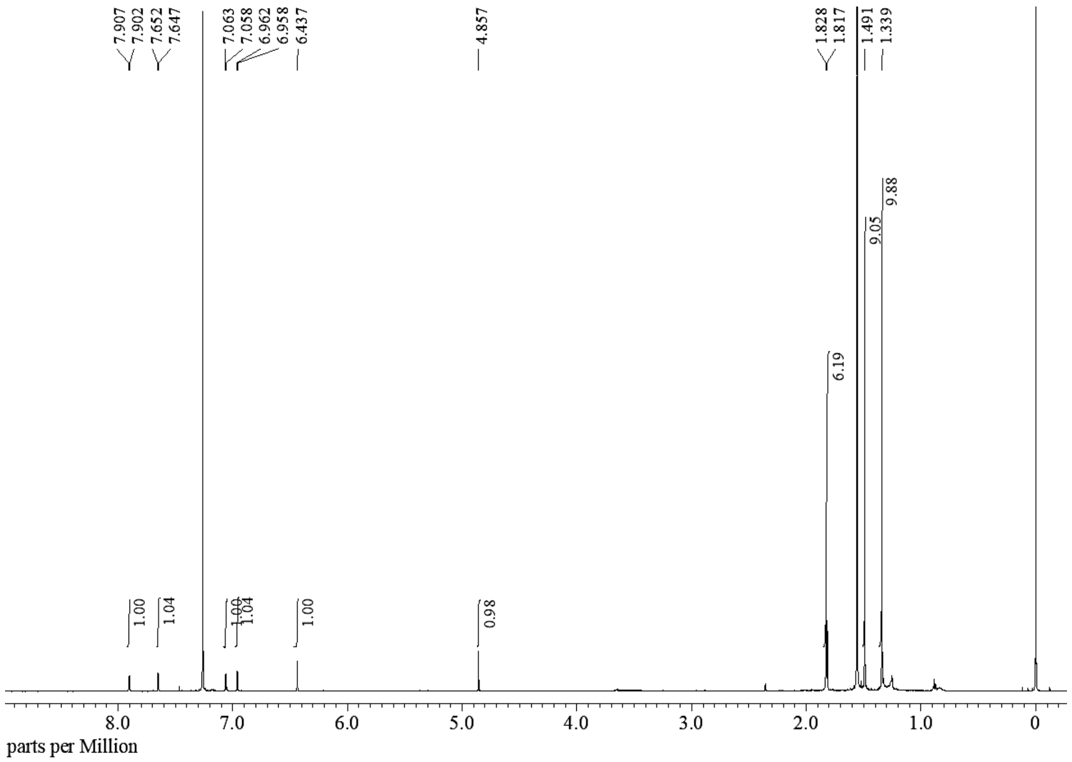

Supplementary Fig. 12. ¹H NMR spectrum of **X70F** in $CDCl_3$ at room temperature.

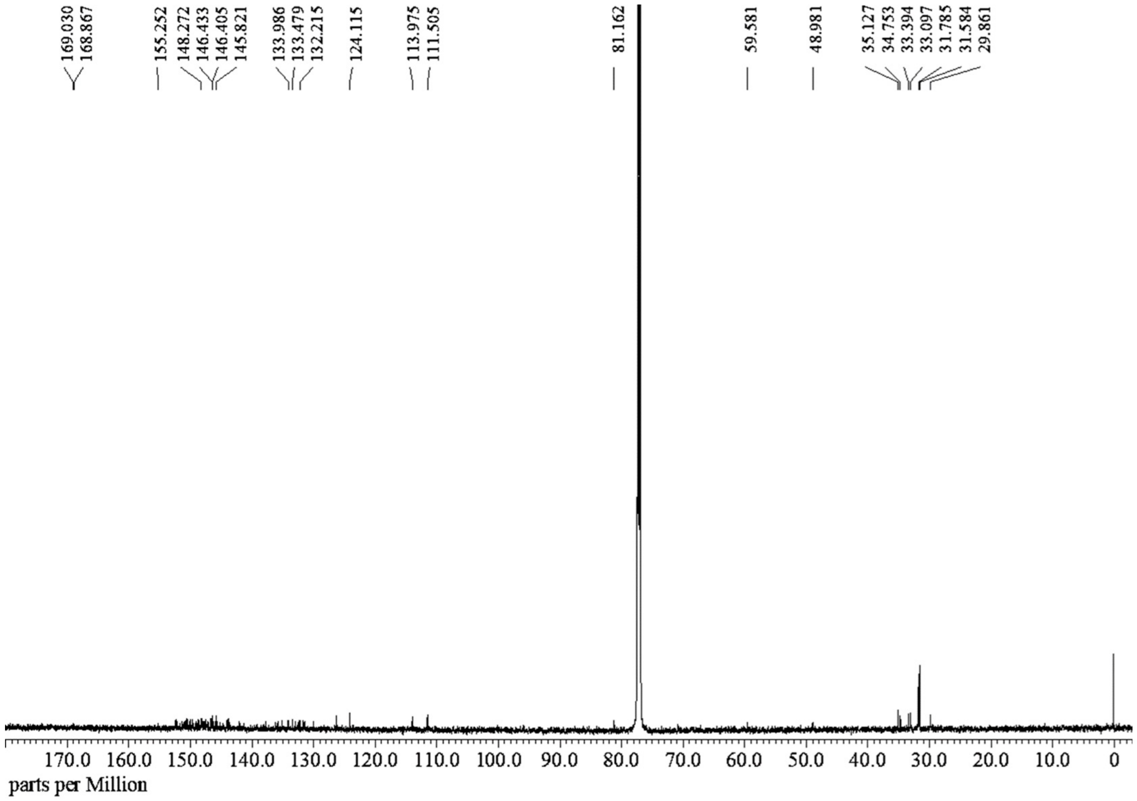

Supplementary Fig. 13. ¹³C NMR spectrum of **X70F** in $CDCl_3$ at room temperature.

Supplementary Table 1. Crystal data and structure refinement for **X70A**.

Empirical formula	$C_{95.33}H_{30}O_{1.33}$
Formula weight	1196.52
Temperature	90 K
Wavelength	0.71073 Å
Crystal system	Trigonal
Space group	*R*-3*c*
Unit cell dimensions	a = 37.451(4) Å
	c = 38.165(4) Å
Volume	46358(10) Å^3
Z	36
Density (calculated)	1.543 g/cm^3
Absorption coefficient	0.090 mm^{-1}
F(000)	22056
Crystal size	0.300 x 0.100 x 0.100 mm^3
Theta range for data collection	1.648 to 23.865°
Index ranges	$-42 \leq h \leq 38$, $-34 \leq k \leq 42$, $-40 \leq l \leq 43$
Reflections collected	71976
Independent reflections	7958 [R(int) = 0.0897]
Completeness to theta = 23.865°	99.8%
Absorption correction	Semi-empirical from equivalents
Max. and min. transmission	0.991 and 0.898
Refinement method	Full-matrix least-squares on F^2
Data / restraints / parameters	7958 / 1163 / 1329
Goodness-of-fit on F^2	1.031
Final R indices [$I > 2\sigma(I)$]	R_1 = 0.0909, wR_2 = 0.2371
R indices (all data)	R_1 = 0.1712, wR_2 = 0.3094
Extinction coefficient	n/a
Largest diff. peak and hole	0.646 and –0.413 e.Å^{-3}

Supplementary Table 2. Crystal data and structure refinement for **X70B**.

Empirical formula	$C_{93}H_{30}O$	
Formula weight	1163.17	
Temperature	90 K	
Wavelength	0.71073 Å	
Crystal system	Monoclinic	
Space group	*C*2/*c*	
Unit cell dimensions	a = 34.25(2) Å	
	b = 14.971(9) Å	β = 99.407(11)°
	c = 21.509(14) Å	
Volume	10881(12) Å^3	
Z	8	
Density (calculated)	1.420 g/cm^3	
Absorption coefficient	0.082 mm^{-1}	
F(000)	4768	
Crystal size	0.100 x 0.100 x 0.010 mm^3	
Theta range for data collection	1.205 to 23.766°	
Index ranges	$-36 \le h \le 38$, $-16 \le k \le 15$, $-24 \le l \le 24$	
Reflections collected	25322	
Independent reflections	8243 [R(int) = 0.2046]	
Completeness to theta = 23.766°	99.3%	
Absorption correction	Semi-empirical from equivalents	
Max. and min. transmission	0.999 and 0.787	
Refinement method	Full-matrix least-squares on F^2	
Data / restraints / parameters	8243 / 12 / 855	
Goodness-of-fit on F^2	0.954	
Final R indices [$I > 2\sigma(I)$]	R_1 = 0.0960, wR_2 = 0.1336	
R indices (all data)	R_1 = 0.2600, wR_2 = 0.1671	
Extinction coefficient	n/a	
Largest diff. peak and hole	0.318 and −0.259 e.Å^{-3}	

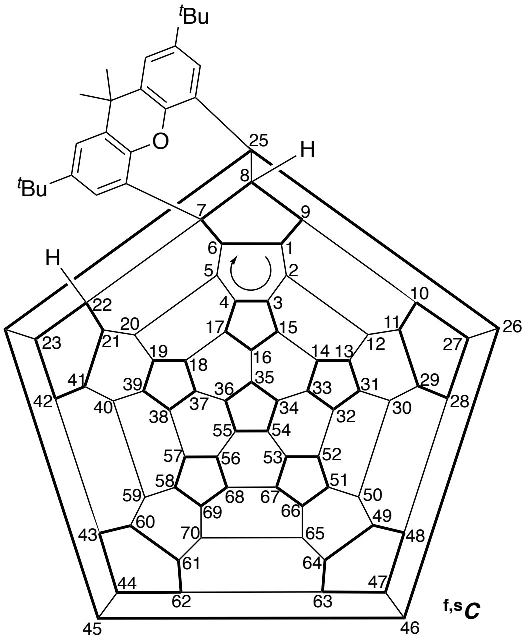

X70A1

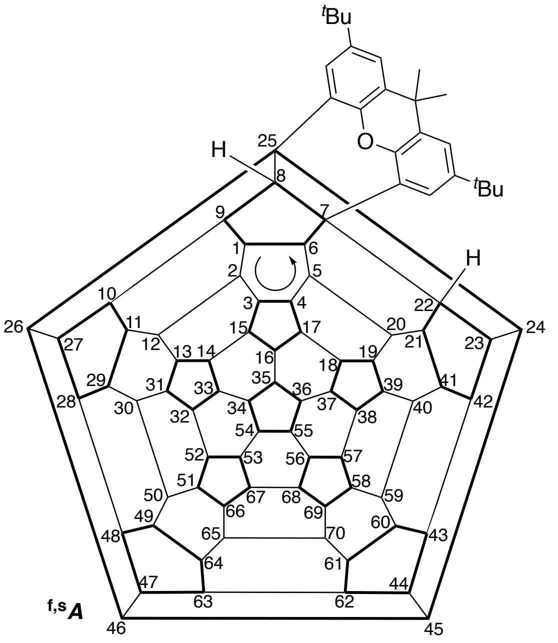

X70A2

X70A1:

($^{f,s}C$)-7,25-(2′,7′-di-*t*-butyl-9′,9′-dimethyl-[4′,5′]xantheno)-7,8,22,25-tetrahydro(C_{70}-D_{5h})[5,6]fullerene

X70A2:

($^{f,s}A$)-7,25-(2′,7′-di-*t*-butyl-9′,9′-dimethyl-[4′,5′]xantheno)-7,8,22,23-tetrahydro(C_{70}-D_{5h})[5,6]fullerene

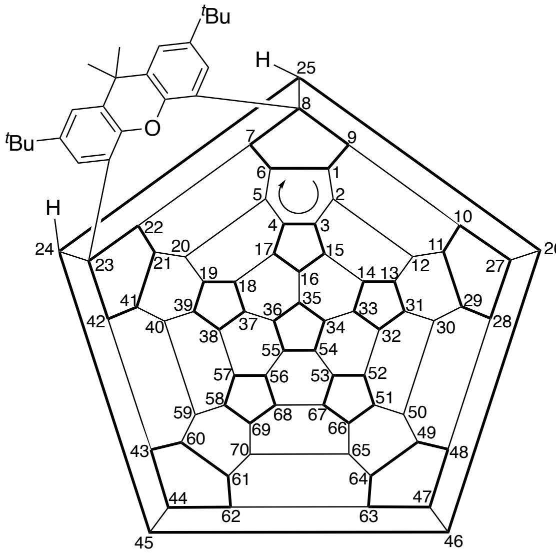

X70B

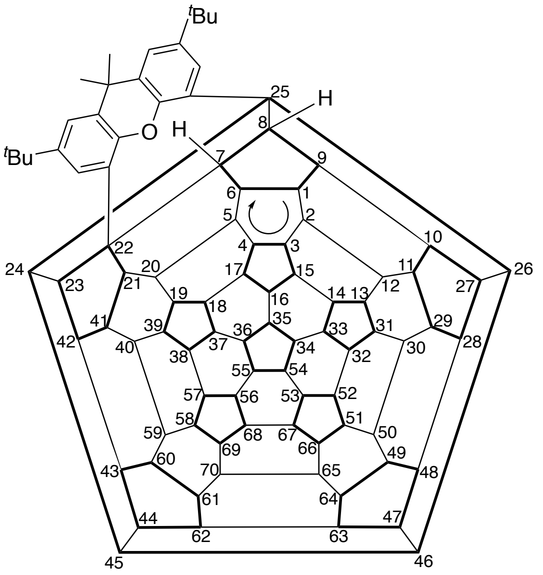

X70C

X70B: 8,23-(2′,7′-di-*t*-butyl-9′,9′-dimethyl-[4′,5′]xantheno)-8,23,24,25-tetrahydro(C_{70}-D_{5h})[5,6]fullerene

X70C, which has been removed from the main text:

22,25-(2′,7′-di-*t*-butyl-9′,9′-dimethyl-[4′,5′]xantheno)-7,8,22,25-tetrahydro(C_{70}-D_{5h})[5,6]fullerene

(continued)

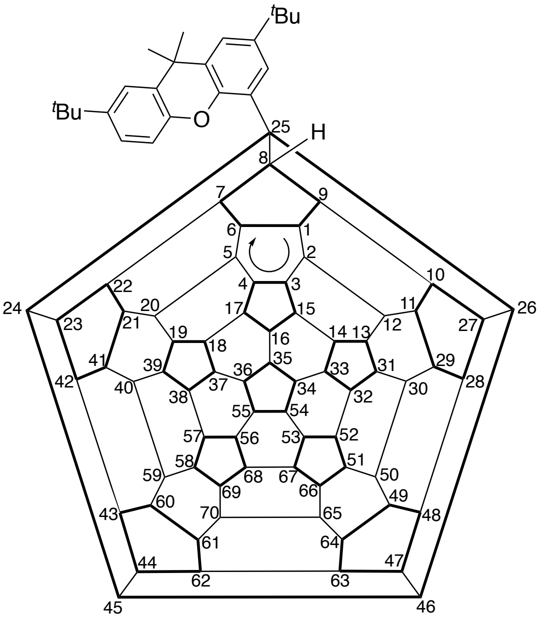

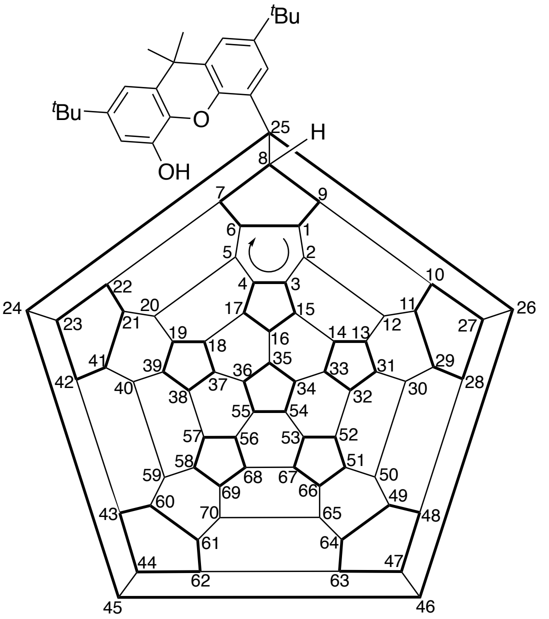

X70D **X70E**

X70D: 25-(2′,7′-di-*t*-butyl-9′,9′-dimethylxanthen-4′-yl)-8,25-dihydro(C_{70}-D_{5h})[5,6]fullerene

X70E is speculated to be

25-(2′,7′-di-*t*-butyl-5′-hydroxy-9′,9′-dimethylxanthen-4′-yl)-8,25-dihydro(C_{70}-D_{5h})[5,6]fullerene

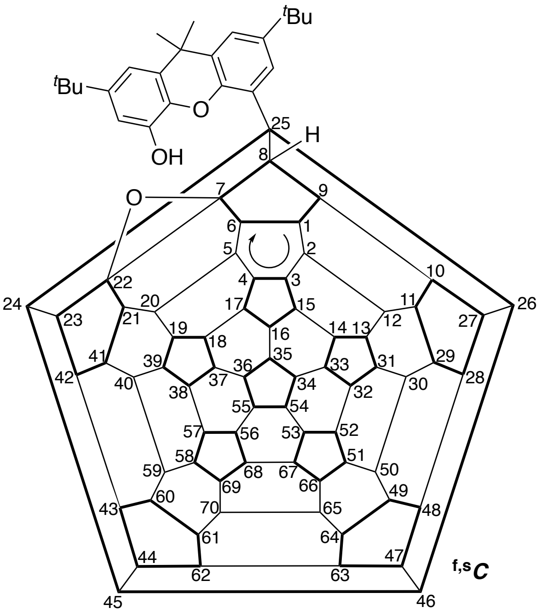

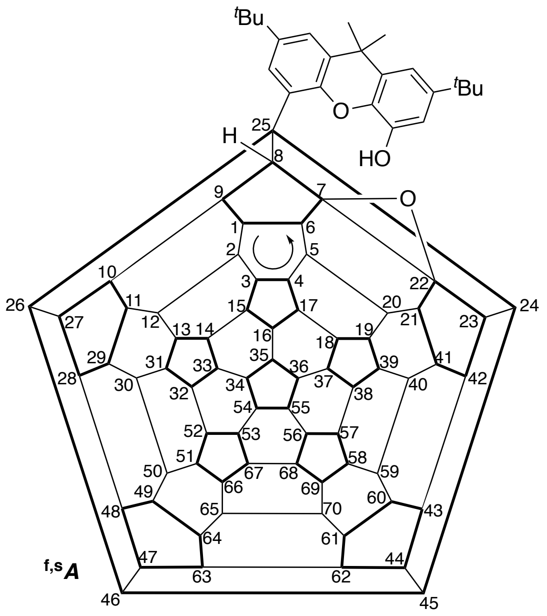

X70F2 **X70F1**

X70F1:

(f,s*A*)-25-(2′,7′-di-*t*-butyl-5′-hydroxy-9′,9′-dimethylxanthen-4′-yl)-7,22-epoxy-7,8,22,25-tetrahydro(C_{70}-D_{5h})[5,6]fullerene

(continued)

X70F2:

(^{f,s}*C*)-25-(2′,7′-di-*t*-butyl-5′-hydroxy-9′,9′-dimethylxanthen-4′-yl)-7,22-epoxy-7,8,22,25-tetrahydro(C_{70}-D_{5h})[5,6]fullerene

Supplementary Fig. 14. Schlegel diagrams and the full names of **X70A-F** with systematic numbering recommended by IUPAC.

Supplementary Table 3. Crystal data and structure refinement for **X70F**

Empirical formula	$C_{107}H_{46}O_3$	
Formula weight	1379.44	
Temperature	90 K	
Wavelength	0.71073 Å	
Crystal system	Monoclinic	
Space group	$P2_1/c$	
Unit cell dimensions	a = 13.918(5) Å	
	b = 16.072(5) Å	β = 94.922(5)°
	c = 27.484(9) Å	
Volume	6125(4) $Å^3$	
Z	4	
Density (calculated)	1.496 g/cm^3	
Absorption coefficient	0.089 mm^{-1}	
F(000)	2848	
Crystal size	0.100 x 0.100 x 0.050 mm^3	
Theta range for data collection	1.487 to 24.292°	
Index ranges	$-16 \leq h \leq 14$, $-18 \leq k \leq 12$, $-31 \leq l \leq 26$	
Reflections collected	30179	
Independent reflections	9840 [R(int) = 0.2051]	
Completeness to theta = 24.292°	99.0%	
Absorption correction	Semi-empirical from equivalents	
Max. and min. transmission	0.996 and 0.752	
Refinement method	Full-matrix least-squares on F^2	
Data / restraints / parameters	9840 / 42 / 1037	
Goodness-of-fit on F^2	1.050	
Final R indices [$I > 2\sigma(I)$]	R_1 = 0.0941, wR_2 = 0.1980	
R indices (all data)	R_1 = 0.2589, wR_2 = 0.2701	
Extinction coefficient	n/a	
Largest diff. peak and hole	0.606 and –0.440 e.$Å^{-3}$	

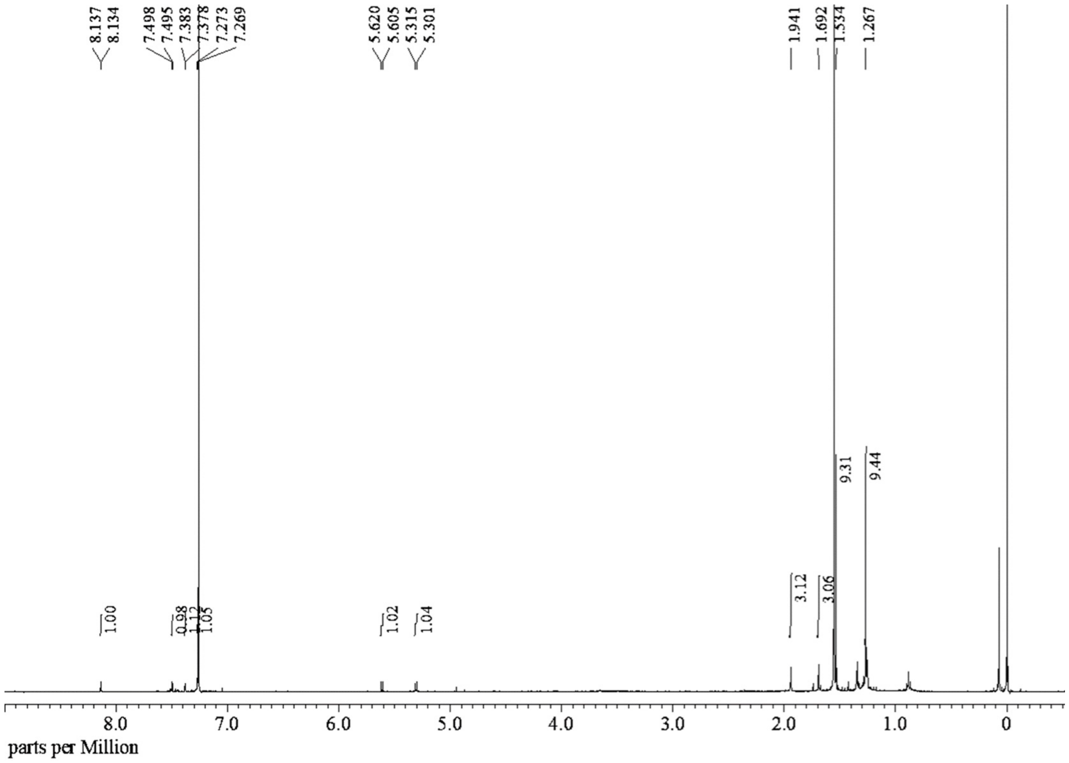

Supplementary Fig. 15. ^{1}H NMR spectrum in $CDCl_3$ at room temperature and the plausible structure of **X70C**.

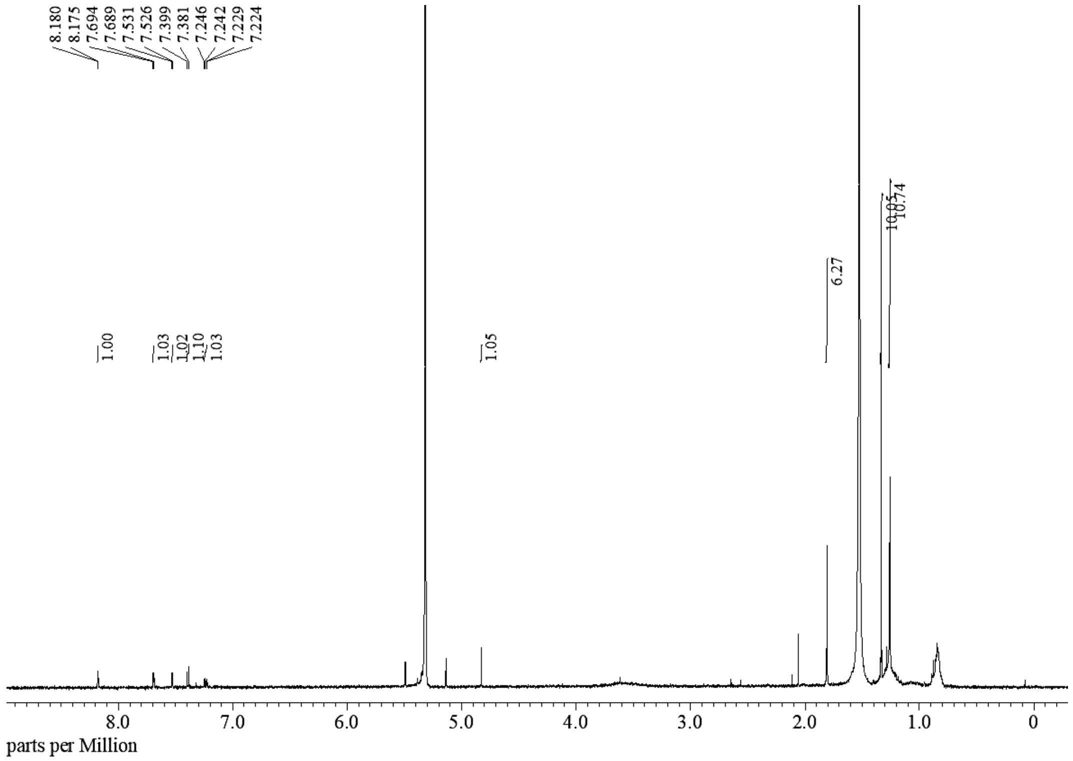

Supplementary Fig. 16. ^{1}H NMR spectrum in CD_2Cl_2 at room temperature and the plausible structure of **X70D**.

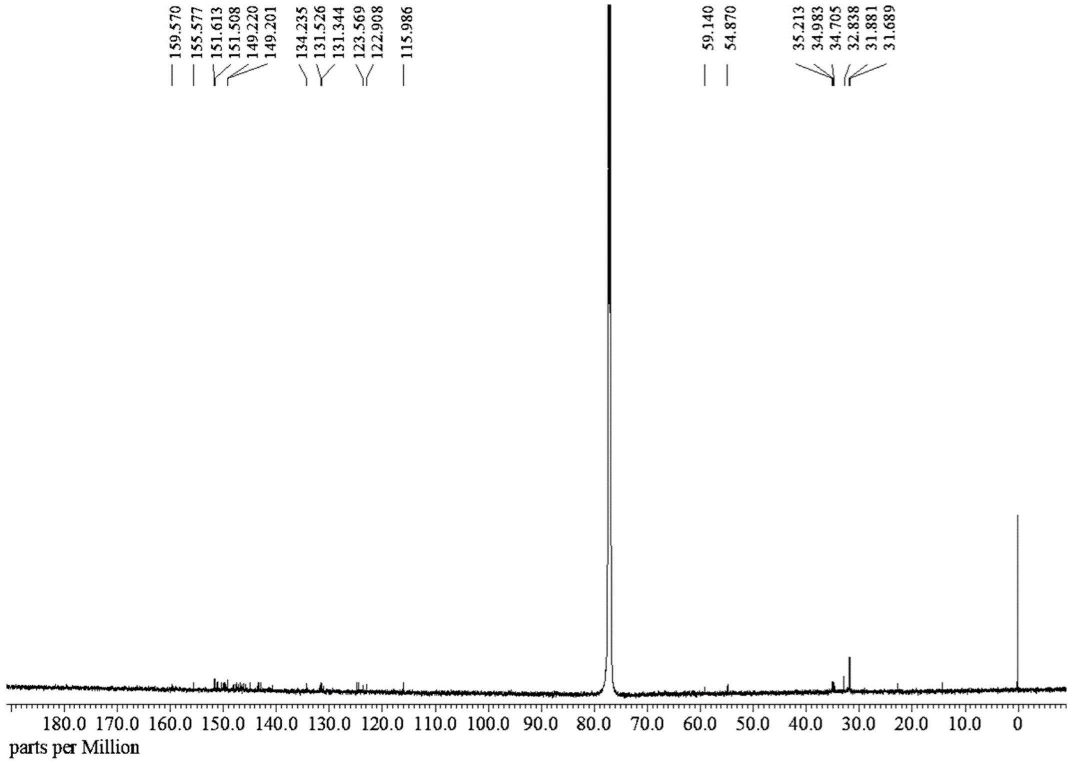

Supplementary Fig. 17. ^{13}C NMR spectrum of **X70D** in $CDCl_3$ at room temperature.

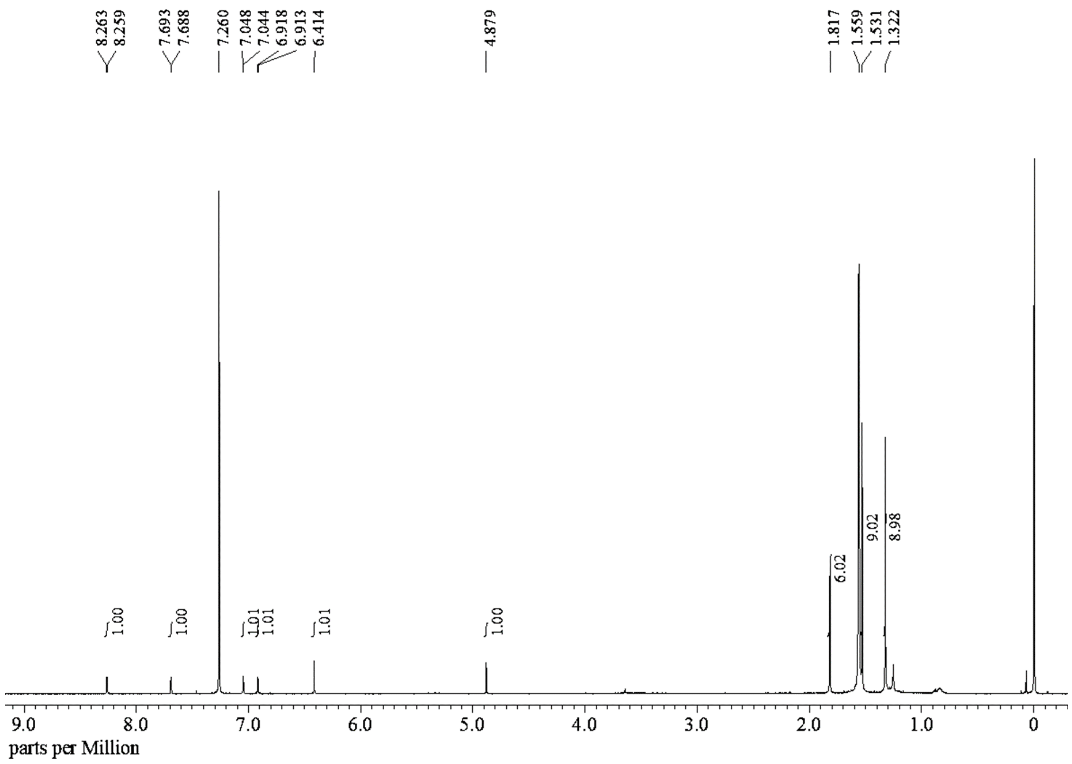

Supplementary Fig. 18. ^{1}H NMR spectrum in $CDCl_3$ at room temperature and the plausible structure of **X70E**.

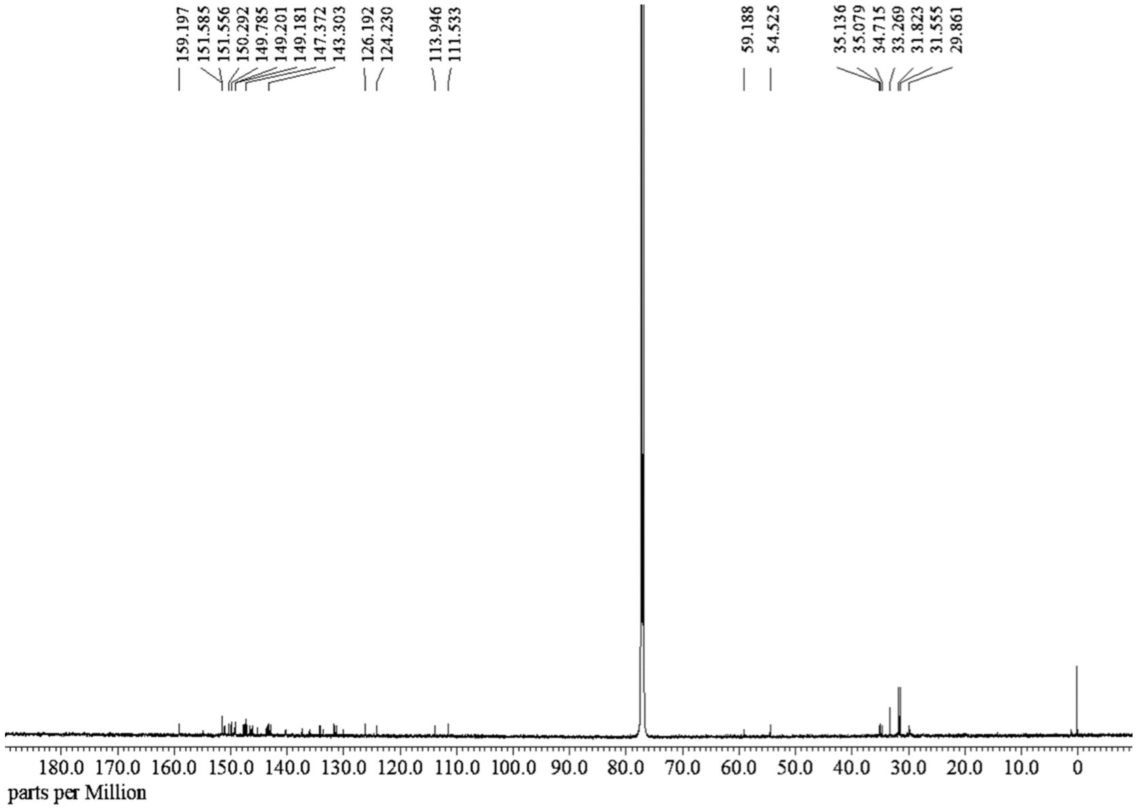

Supplementary Fig. 19. ^{13}C NMR spectrum of **X70E** in $CDCl_3$ at room temperature.

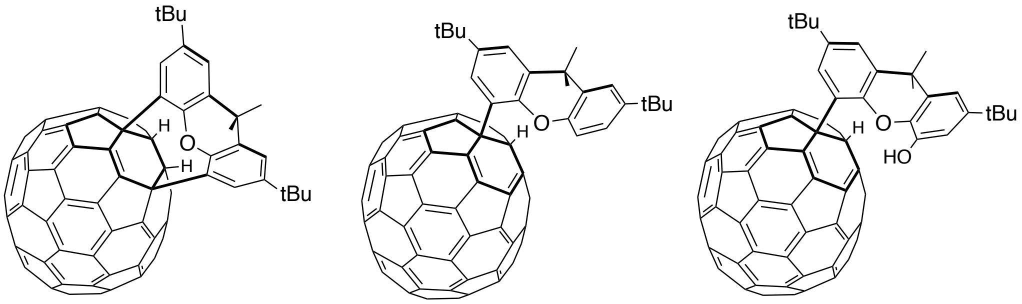

X70C **X70D** **X70E**

Supplementary Fig. 20. The plausible structures for **X70C**, **X70D** and **X70E**.

Supplementary Table 4. Photophysical data of C_{70}, **X70A** and **X70F**

compound	λ_{abs} [nm] ($\log\varepsilon$)	$^{*}\lambda_{em}$ [nm]
C_{70}	334 (4.61), 381 (4.63), 472 (4.42), 533 (4.10), 597 (3.7), 645 (3.2)	667, 694, 723
**X70A**	361 (4.31), 401 (4.34), 533 (3.85), 579 (3.69), 632 (3.47)	681, 699, 736
**X70F**	399 (4.30), 455 (4.20), 623 (3.37)	676, 731

* λ_{ex} = 500 nm

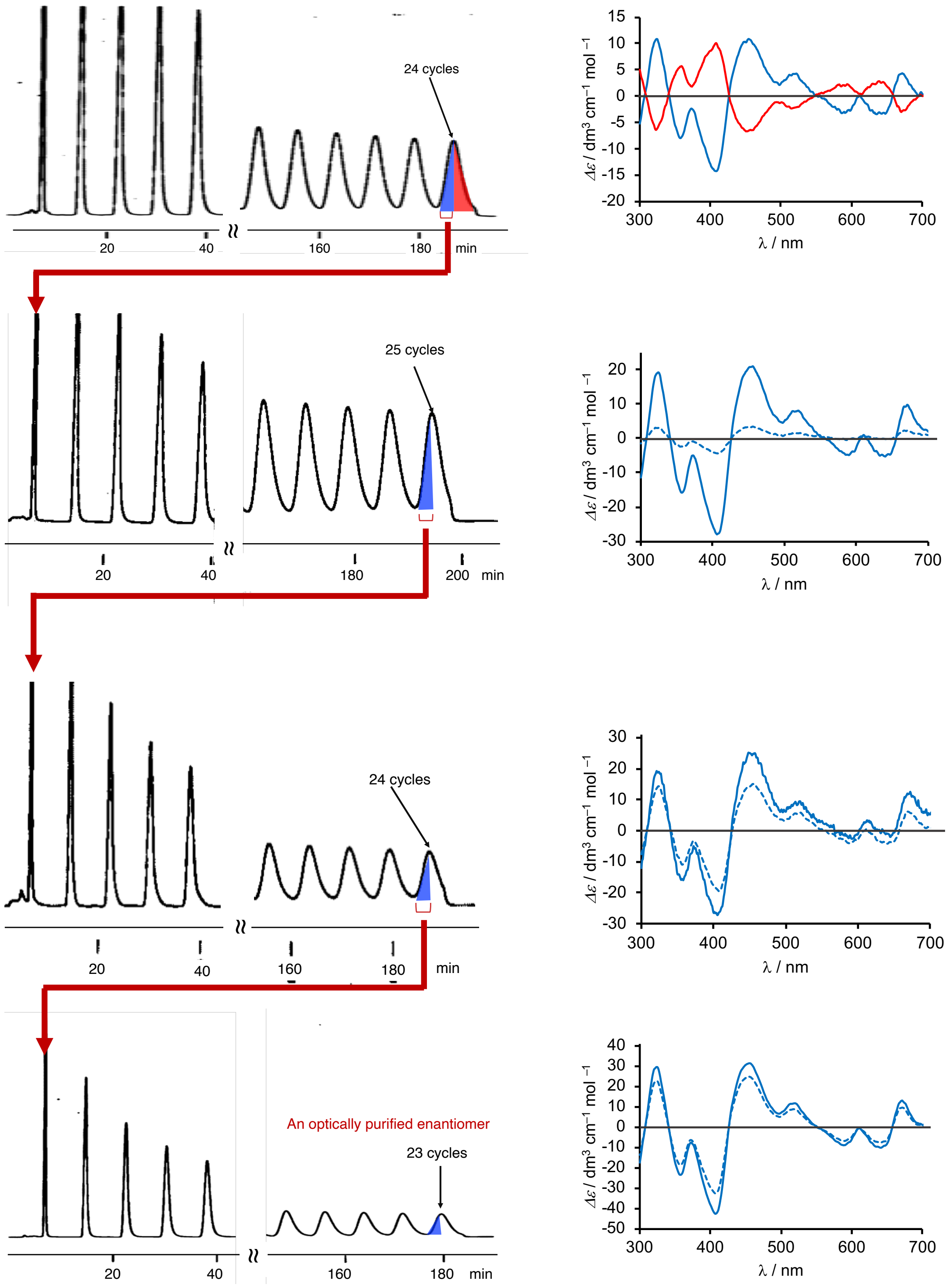

Supplementary Fig. 21. Preparative chiral HPLC profiles of **X70A** monitored by a UV-Vis detector at 326 nm. A solution of **X70A** was subjected on a ϕ10 x 250 mm Cholester column (Nacalai Tesque) with hexane / *i*PrOH (4/1 *v*/*v*) as eluent at a flow rate of 4.5 mL min^{-1}, where the former (solid line) and latter (dotted line) half of the 23-25 cycled elution peaks were collected and checked by CD spectra.

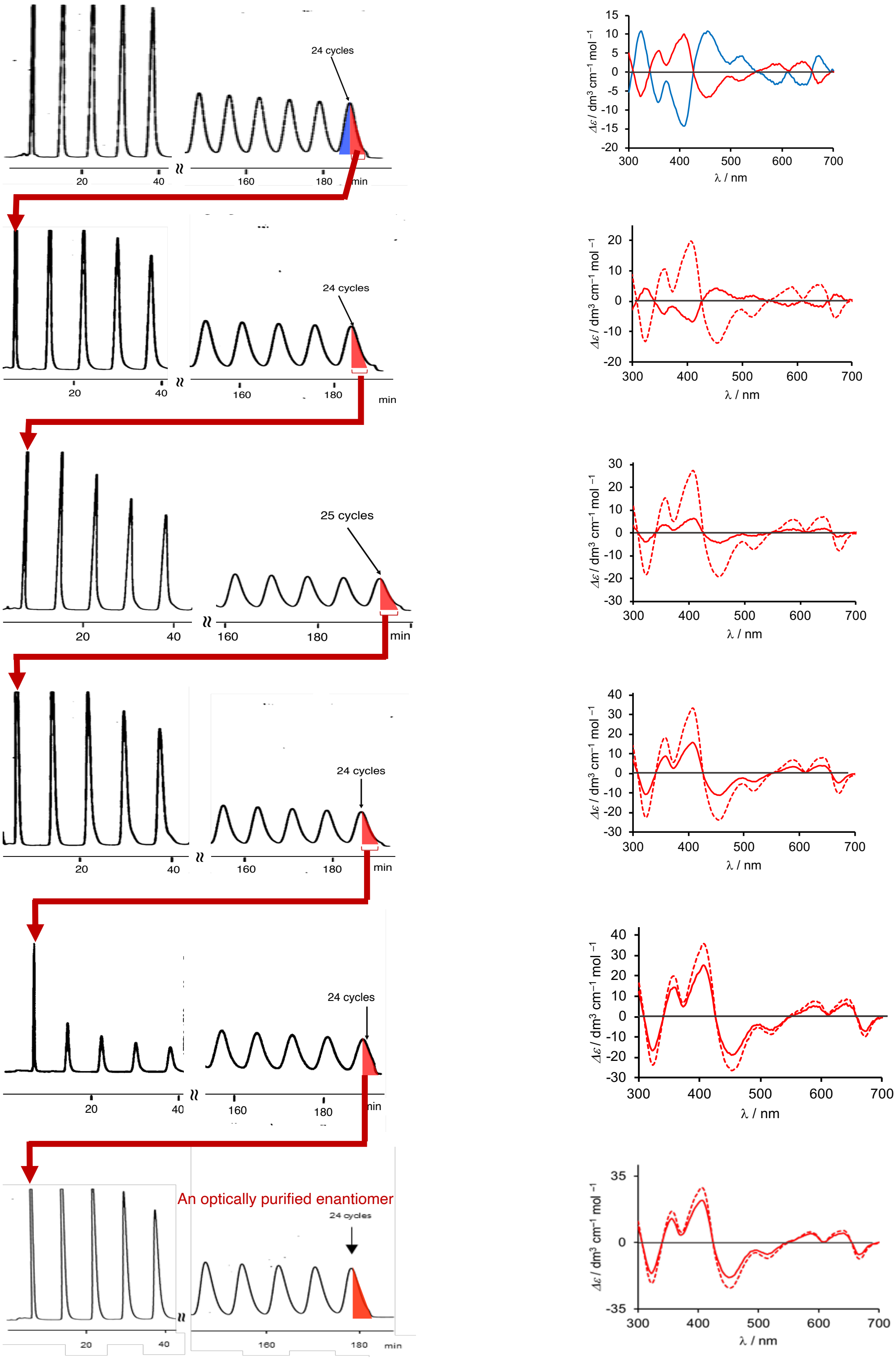

Supplementary Fig. 22. Preparative chiral HPLC profiles of **X70A** monitored by a UV-Vis detector at 326 nm. A solution of **X70A** was subjected on a ϕ10 x 250 mm Cholester column (Nacalai Tesque) with hexane / *i*PrOH (4/1 *v*/*v*) as eluent at a flow rate of 4.5 mL min^{-1}, where the former (solid line) and latter (dotted line) half of the 24 or 25 cycled elution peaks were collected and checked by CD spectra.

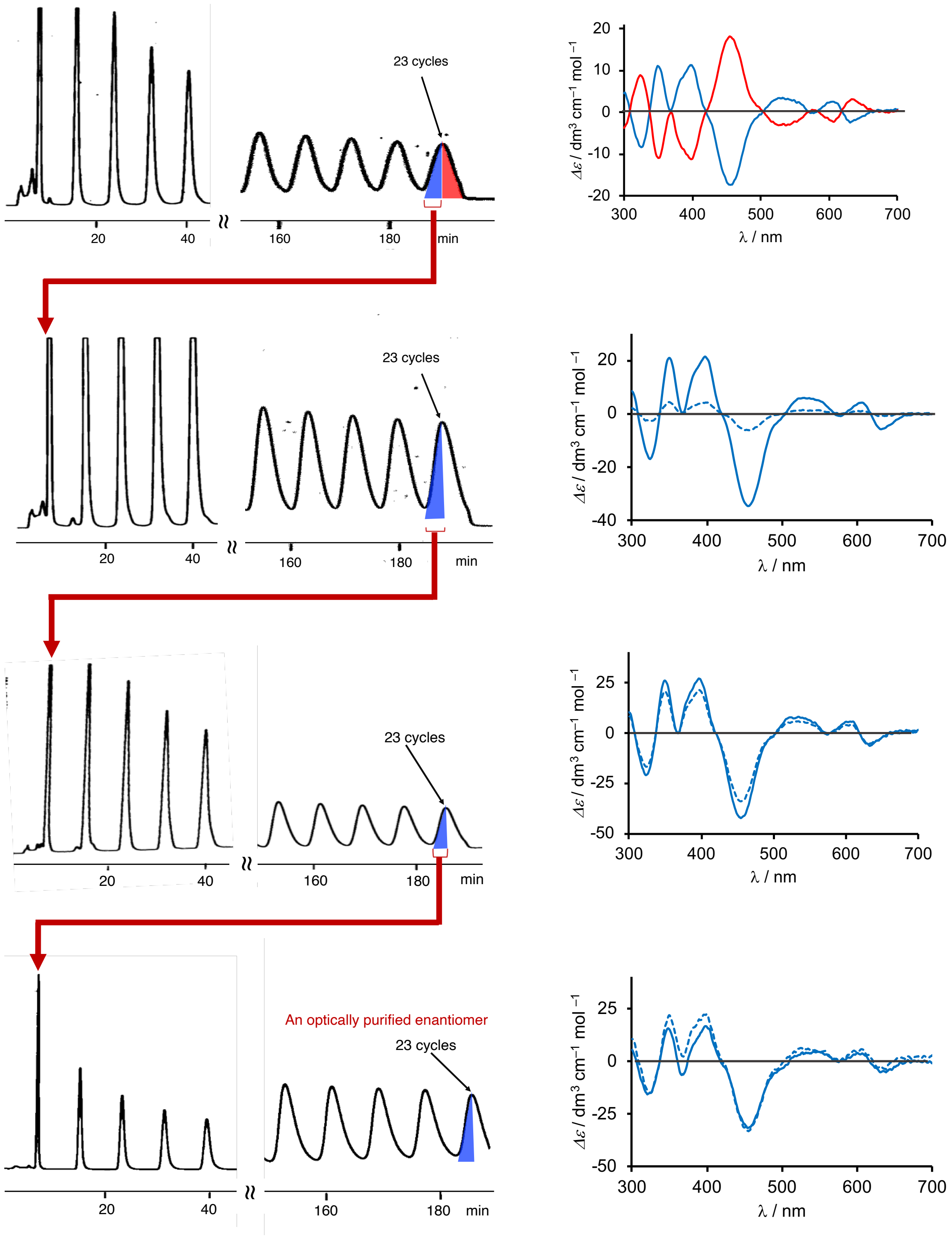

Supplementary Fig. 23. Preparative chiral HPLC profiles of **X70F** monitored by a UV-Vis detector at 326 nm. A solution of **X70F** was subjected on a ϕ10 x 250 mm Cholester column (Nacalai Tesque) with hexane / *i*PrOH (4/1 *v*/*v*) as eluent at a flow rate of 4.5 mL min^{-1}, where the former (solid line) and latter (dotted line) half of the 23 cycled elution peaks were collected and checked by CD spectra.

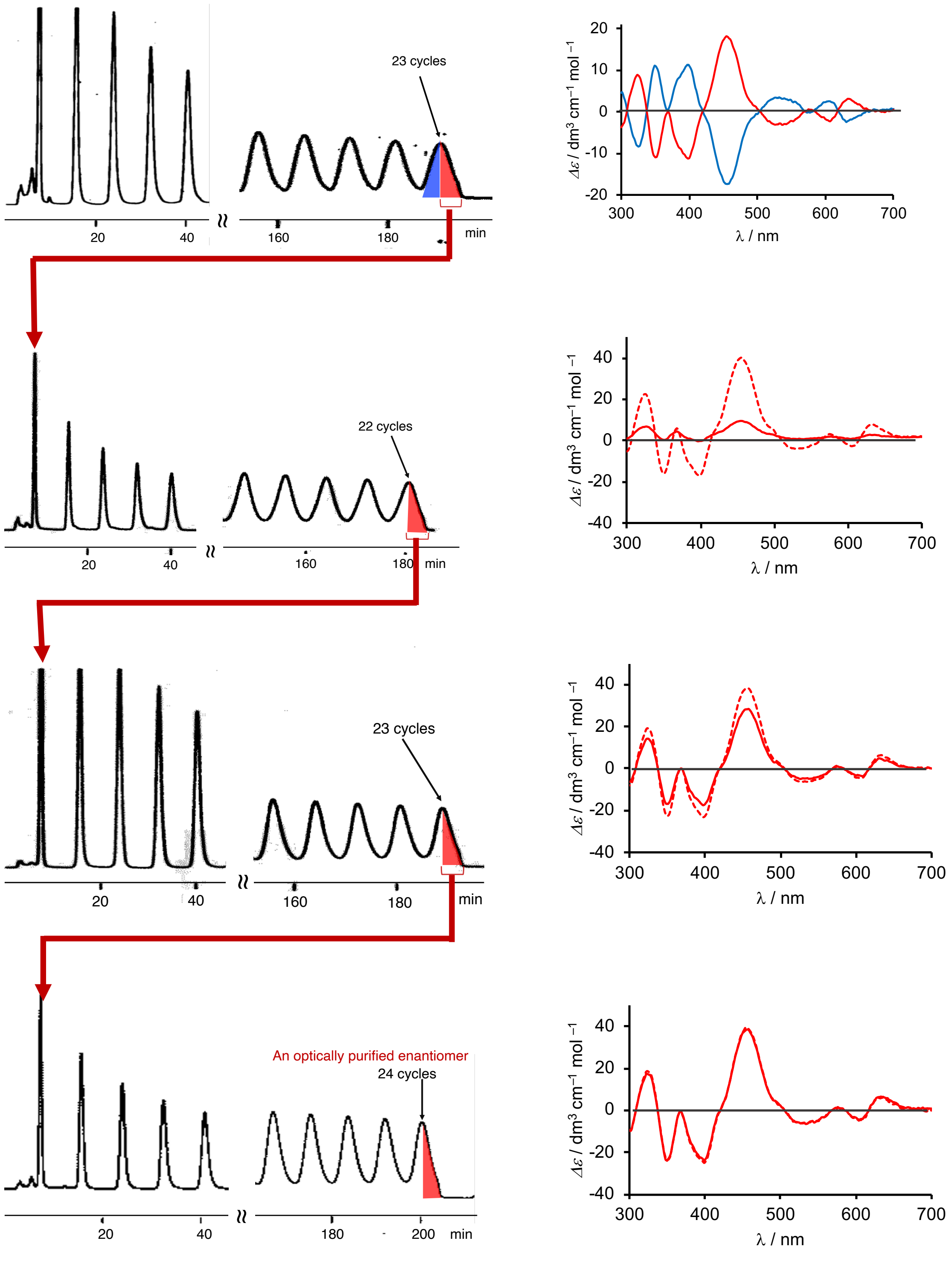

Supplementary Fig. 24. Preparative chiral HPLC profiles of **X70F** monitored by a UV-Vis detector at 326 nm. A solution of **X70F** was subjected on a *ϕ*10 x 250 mm Cholester column (Nacalai Tesque) with hexane / *i*PrOH (4/1 *v*/*v*) as eluent at a flow rate of 4.5 mL min^{-1}, where the former (solid line) and latter (dotted line) half of the 22-24 cycled elution peaks were collected and checked by CD spectra.

Computational Details

Computational Details

The geometrical optimizations of **X70A** and **X70F** were conducted without any symmetry constraints by density functional theory (DFT) calculations using the B3LYP combinations in the Gaussian 16 computational chemistry program[S3]. The 6-31G* basis set was applied for the C, O, and H atoms according to the precedent of DFT calculations for chiral fullerenes[S4]. The electronic excitation energies, oscillator strengths, and rotational strengths of the 100 lowest energy electronic excitations for the most stable conformers of **X70A** and **X70F** were calculated at the TDB3LYP/6-31G* level of theory. In the comparison of the calculated CD spectra with experimental spectra, Gaussian band shapes with bandwidths of 0.19 and 0.23 eV were used to simulate the CD spectra for **X70A2** and **X70F2**, respectively, and this produced the best similarity factors using the SpecDis software package (Version 1.71)[S5].

Calculation results

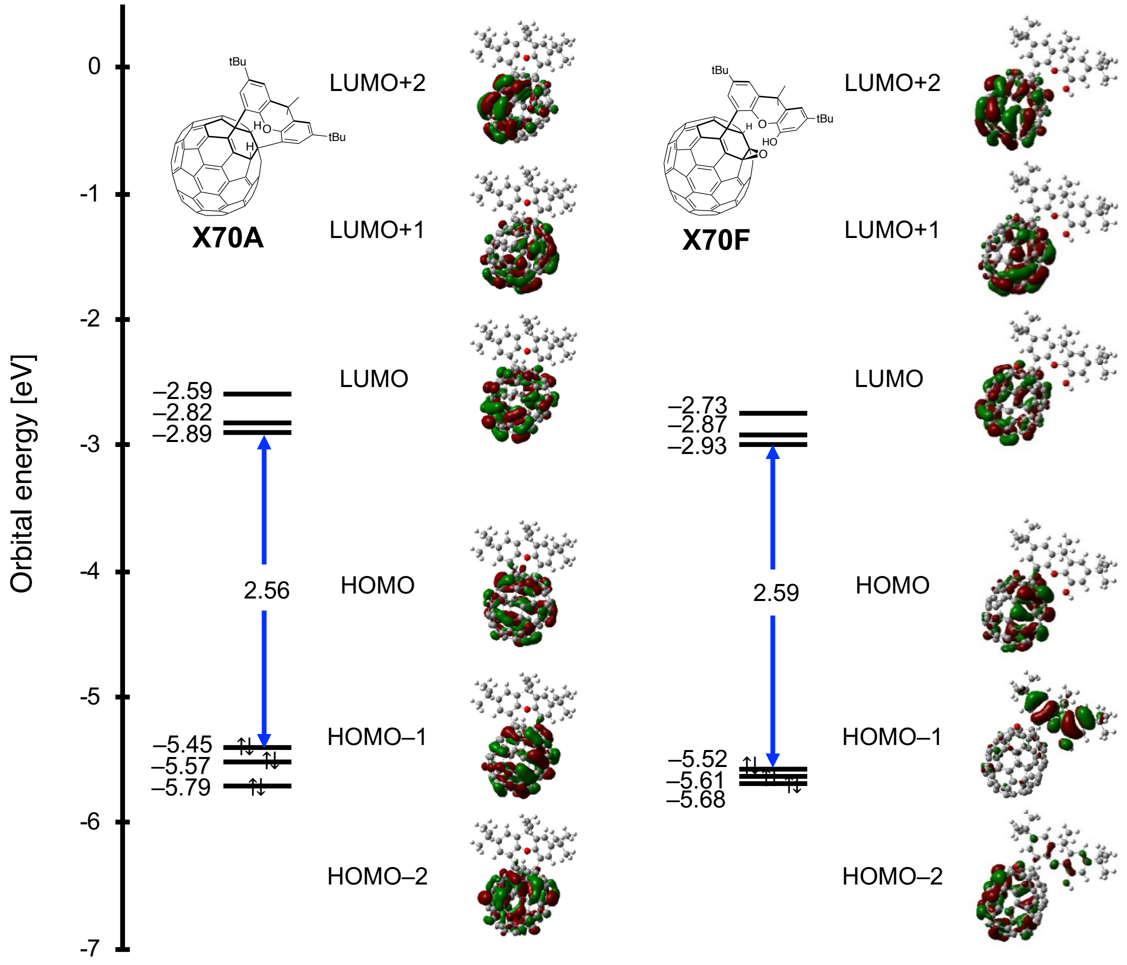

Supplementary Fig. 25. MO diagrams of **X70A** and **X70F** calculated at the B3LYP/6-31G(d) level.

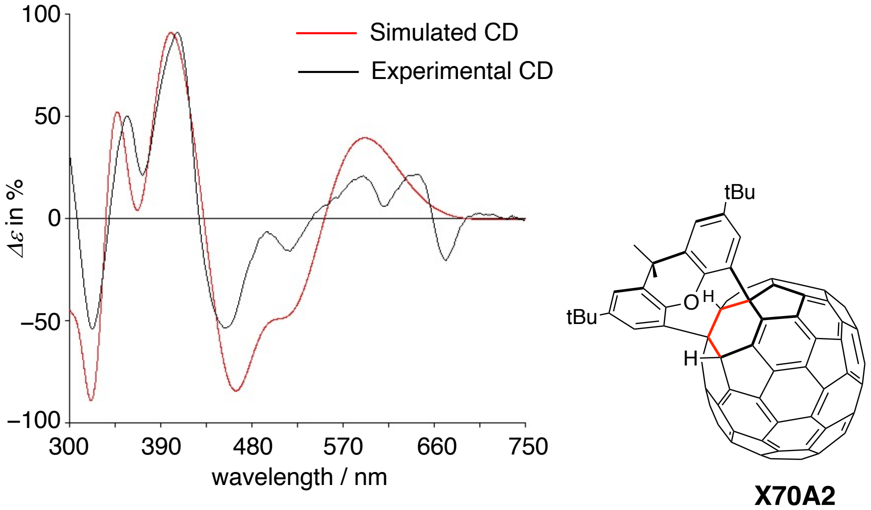

X70A2

Supplementary Fig. 26. The CD spectrum of the second peak of **X70A** (black) in toluene and the simulated CD spectrum of the enantiomer calculated from the structure shown above based on the TD-DFT method and using the SpecDis software package (red). From the obtained result, we assigned the absolute structure of **X70A2** as ($^{f,s}A$)-7,25-xantheno-7,8,22,23-tetrahydro(C_{70}-D_{5h})[5,6]fullerene with a similarity factor of 0.89.

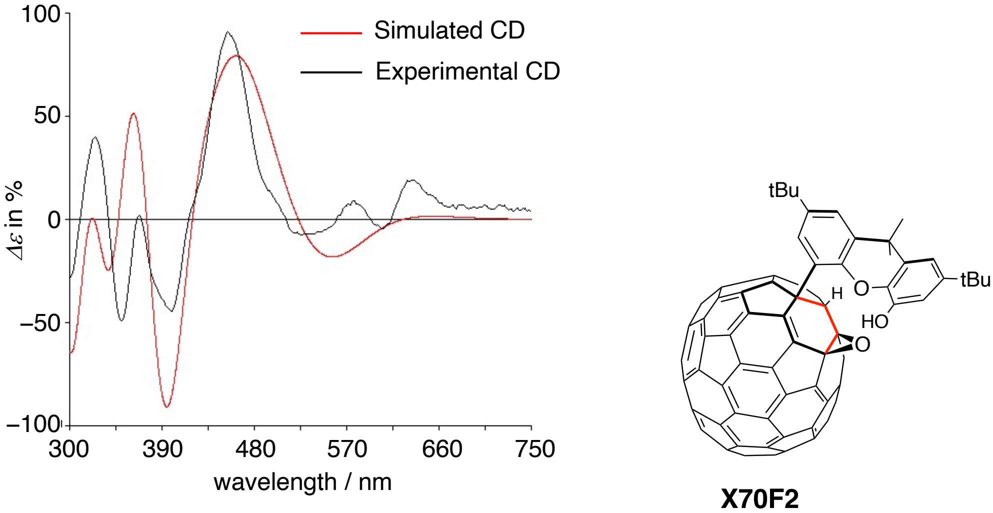

X70F2

Supplementary Fig. 27. The CD spectrum of the second peak of **X70F** (black) in toluene and the simulated CD spectrum of the enantiomer calculated from the structure shown above based on the TD-DFT method and using the SpecDis software package (red). From the obtained result, we assigned the absolute structure of **X70F2** as ($^{f,s}C$)-25-(5'-hydroxyxanthenyl)-7,22-epoxy-7,8,22,25-tetrahydro(C_{70}-D_{5h})[5,6]fullerene with a similarity factor of 0.81.

Supplementary Table 5. Representative data from TD-DFT calculations of **X70A**

Ground to excited state transition electric dipole moments (Au):

state	X	Y	Z	Dip. S.	Osc.
1	–0.0146	0.0980	–0.0069	0.0099	0.0005

Ground to excited state transition velocity dipole moments (Au):

state	X	Y	Z	Dip. S.	Osc.
1	0.0010	–0.0056	0.0008	0.0000	0.0003

Ground to excited state transition magnetic dipole moments (Au):

state	X	Y	Z
1	–1.5290	0.8626	–0.2115

Ground to excited state transition velocity quadrupole moments (Au):

state	XX	YY	ZZ	XY	XZ	YZ
1	–0.0568	–0.0459	0.0853	–0.1448	0.1322	0.1089

<0|del|b> * <b|rxdel|0> + <0|del|b> * <b|delr+rdel|0>

Rotatory Strengths (R) in cgs (10**-40 erg-esu-cm/Gauss)

state	XX	YY	ZZ	R(velocity)	E-M Angle
1	–20.8512	–6.9825	-34.3465	–20.7267	129.93

1/2[<0|r|b>*<b|rxdel|0> + (<0|rxdel|b>*<b|r|0>)*]

Rotatory Strengths (R) in cgs (10**-40 erg-esu-cm/Gauss)

state	XX	YY	ZZ	R(length)
1	–15.8105	–59.7508	–1.0266	-25.5293

1/2[<0|del|b>*<b|r|0> + (<0|r|b>*<b|del|0>)*] (Au)

state	X	Y	Z	Dip. S.	Osc.(frdel)
1	–0.0000	–0.0006	–0.0000	0.0006	0.0004

Excitation energies and oscillator strengths:

Excited State 1: Singlet-A 2.0267 eV 611.75 nm f=0.0005 <S**2>=0.000

298 -> 299 0.69628

Supplementary Table 6. Representative data from TD-DFT calculations of **X70F**

Ground to excited state transition electric dipole moments (Au):

state	X	Y	Z	Dip. S.	Osc.
1	0.2049	0.1567	0.0666	0.0710	0.0036

Ground to excited state transition velocity dipole moments (Au):

state	X	Y	Z	Dip. S.	Osc.
1	–0.0141	–0.0106	–0.0057	0.0003	0.0031

Ground to excited state transition magnetic dipole moments (Au):

state	X	Y	Z
1	0.2869	–0.0397	–0.6763

Ground to excited state transition velocity quadrupole moments (Au):

state	XX	YY	ZZ	XY	XZ	YZ
1	–0.0122	0.0971	–0.1157	–0.1075	–0.0936	0.0109

<0|del|b> * <b|rxdel|0> + <0|del|b> * <b|delr+rdel|0>

Rotatory Strengths (R) in cgs (10**-40 erg-esu-cm/Gauss)

state	XX	YY	ZZ	R(velocity)	E-M Angle
1	18.3840	–4.4616	–11.6396	0.7609	88.98

1/2[<0|r|b>*<b|rxdel|0> + (<0|rxdel|b>*<b|r|0>)*]

Rotatory Strengths (R) in cgs (10**-40 erg-esu-cm/Gauss)

state	XX	YY	ZZ	R(length)
1	–41.5688	4.3992	31.8695	–1.7667

1/2[<0|del|b>*<b|r|0> + (<0|r|b>*<b|del|0>)*] (Au)

state	X	Y	Z	Dip. S.	Osc.(frdel)
1	–0.0029	–0.0017	–0.0004	0.0049	0.0033

Excitation energies and oscillator strengths:

Excited State 1: Singlet-A 2.0440 eV 606.59 nm f=0.0036 <S**2>=0.000

306 -> 307 0.68175

306 -> 308 –0.11452

Supplementary Table 7. **Cartesian Coordinates**

The cartesian coordinates (in Å) optimized at the B3LYP/6-31G(d) level are shown.

X70A

O	4.221	5.852	2.893
C	2.253	7.242	4.058
H	3.125	7.709	4.206
C	1.93	7.371	2.492
C	0.709	8.355	2.285
C	0.129	9.109	3.228
C	0.422	8.889	4.642
C	1.254	7.894	4.994
C	1.003	7.123	6.143
C	1.504	5.801	5.927
C	2.416	5.743	4.702
C	2.042	4.532	3.785
H	2.88	4.014	3.617
C	1.488	4.934	2.452
C	1.427	6.149	1.874
C	0.244	6.544	0.982
C	-0.14	7.876	1.291
C	-1.487	8.16	1.174
C	-1.996	8.962	2.213
C	-1.293	9.423	3.25
C	-1.794	9.432	4.554
C	-0.764	9.108	5.415
C	-1.057	8.435	6.611
C	-0.133	7.397	7.003
C	-0.592	6.288	7.778
C	-0.166	4.961	7.485
C	0.811	4.706	6.448
C	0.579	3.531	5.594
C	1.022	3.58	4.25
C	0.19	3.119	3.192
C	0.48	3.946	2.057
C	-0.526	4.245	1.142
C	-0.606	5.548	0.628
C	-2.037	6.029	0.552
C	-2.522	7.253	0.845
C	-3.673	7.516	1.62

C	-3.379	8.614	2.462
C	-3.953	8.679	3.737
C	-3.147	9.145	4.87
C	-3.423	8.634	6.135
C	-2.381	8.315	7.007
C	-2.811	7.19	7.763
C	-1.939	6.258	8.179
C	-2.364	4.881	8.058
C	-1.254	4.061	7.673
C	-1.546	2.972	6.887
C	-0.645	2.763	5.788
C	-1.438	2.24	4.697
C	-1.112	2.538	3.342
C	-2.154	2.934	2.406
C	-1.83	3.784	1.331
C	-2.763	4.84	0.971
C	-4.039	5.028	1.597
C	-4.518	6.398	1.944
C	-5.281	6.61	3.133
C	-5.054	7.773	3.997
C	-5.314	7.304	5.32
C	-4.55	7.805	6.308
C	-4.215	6.928	7.416
C	-4.552	5.626	7.404
C	-3.663	4.551	7.811
C	-3.922	3.415	6.954
C	-2.859	2.675	6.493
C	-2.822	2.263	5.122
C	-3.795	2.559	4.252
C	-3.473	2.931	2.878
C	-4.419	3.945	2.478
C	-5.294	4.172	3.634
C	-5.702	5.478	3.92
C	-5.748	5.913	5.298
C	-5.405	5.076	6.347
C	-4.972	3.755	6.051
C	-4.929	3.349	4.755
C	3.972	5.694	5.199
C	4.569	5.719	6.464
H	4.035	5.551	7.232

C	5.936	5.984	6.627
C	6.689	6.321	5.497
H	7.598	6.575	5.609
C	6.146	6.296	4.211
C	6.782	6.804	2.927
C	5.671	7.569	2.172
C	5.791	8.797	1.505
H	6.654	9.174	1.382
C	4.671	9.483	1.015
C	3.404	8.976	1.294
H	2.635	9.469	1.034
C	3.247	7.759	1.949
C	4.4	7.07	2.262
C	4.833	5.882	4.139
C	6.576	6.018	8.01
C	5.639	5.335	8.996
H	5.484	4.41	8.711
H	4.785	5.816	9.022
H	6.044	5.339	9.888
C	7.954	5.484	7.92
H	8.409	5.609	8.778
H	8.444	5.96	7.217
H	7.92	4.528	7.704
C	6.702	7.469	8.373
H	5.809	7.862	8.462
H	7.197	7.939	7.67
H	7.183	7.552	9.223
C	7.268	5.615	2.07
H	7.922	5.092	2.578
H	7.685	5.953	1.25
H	6.504	5.047	1.837
C	7.993	7.708	3.225
H	7.694	8.506	3.71
H	8.415	7.977	2.382
H	8.641	7.217	3.772
C	4.85	10.815	0.309
C	3.525	11.364	-0.201
H	2.946	11.581	0.56
H	3.086	10.69	-0.762
H	3.688	12.173	-0.729

C	5.383	11.781	1.288
H	5.27	12.691	0.942
H	6.335	11.604	1.437
H	4.897	11.69	2.134
C	5.706	10.634	-0.915
H	5.904	11.51	-1.308
H	5.227	10.083	-1.57
H	6.544	10.191	-0.666

X70F

C	1.863	6.749	25.408
C	0.248	6.689	25.485
C	-0.397	7.356	24.217
C	0.234	7.637	23.01
C	1.696	7.551	22.952
C	2.518	7.228	24.157
C	3.613	8.12	24.417
C	3.673	8.356	25.841
C	2.483	7.647	26.441
C	1.871	8.206	27.484
C	0.351	8.214	27.52
C	-0.313	7.613	26.537
C	-1.468	8.337	26.038
C	-1.491	8.116	24.581
C	-1.974	9.177	23.798
C	-1.295	9.486	22.58
C	-0.186	8.714	22.232
C	0.948	9.373	21.668
C	2.096	8.729	22.098
C	3.329	9.426	22.322
C	4.131	9.118	23.541
C	4.85	10.129	24.162
C	4.914	10.37	25.496
C	4.155	9.473	26.446
C	3.524	10.119	27.541
C	2.31	9.449	28.045
C	1.197	10.206	28.399
C	0.028	9.468	28.119
C	-1.118	10.169	27.626
C	-1.88	9.515	26.578

C	-2.32	10.627	25.685
C	-2.37	10.391	24.401
C	-1.94	11.512	23.453
C	-1.316	10.879	22.413
C	-0.24	11.567	21.742
C	0.896	10.807	21.334
C	2.219	11.387	21.293
C	3.348	10.701	21.797
C	4.254	11.741	22.374
C	4.96	11.464	23.503
C	5.08	12.449	24.521
C	5.014	11.712	25.822
C	4.377	12.321	26.846
C	3.572	11.467	27.728
C	2.439	12.258	28.119
C	1.167	11.671	28.336
C	0.028	12.348	27.843
C	-1.104	11.53	27.468
C	-1.858	11.847	26.29
C	-1.531	12.968	25.469
C	-1.619	12.764	24.009
C	-0.722	13.566	23.207
C	-0.091	12.936	22.062
C	1.267	13.557	21.898
C	2.346	12.811	21.564
C	3.648	13.042	22.221
C	3.733	13.97	23.218
C	4.488	13.656	24.373
C	3.817	14.262	25.523
C	3.768	13.589	26.698
C	2.574	13.518	27.489
C	1.435	14.179	27.027
C	0.153	13.56	27.166
C	-0.647	13.875	25.959
C	0.227	14.704	25.156
C	0.194	14.521	23.716
C	1.383	14.553	22.955
C	2.588	14.769	23.576
C	2.64	14.916	25.036
C	1.462	14.892	25.773

C	-0.191	5.225	25.663
C	-0.915	4.59	24.655
C	-1.319	3.256	24.735
C	-0.911	2.518	25.852
C	-0.151	3.104	26.884
C	0.438	2.274	28.012
C	0.552	3.163	29.239
C	0.505	2.69	30.554
C	0.699	3.518	31.657
C	0.927	4.876	31.43
C	0.946	5.383	30.159
C	0.792	4.516	29.053
C	0.159	4.449	26.777
C	-2.129	2.623	23.59
C	-3.261	3.573	23.155
C	-1.188	2.363	22.421
C	-2.766	1.315	24.004
C	1.844	1.869	27.61
C	-0.385	1.016	28.308
C	0.677	2.896	33.076
C	0.775	3.989	34.149
C	-0.622	2.12	33.306
C	1.853	1.962	33.223
O	0.856	5.106	27.793
O	2.515	6.345	22.963
O	1.164	6.716	29.897
H	2.201	5.823	25.581
H	-1.145	5.088	23.878
H	-1.155	1.602	25.918
H	0.333	1.768	30.701
H	1.073	5.456	32.166
H	-2.874	4.378	22.75
H	-3.795	3.825	23.935
H	-3.832	3.121	22.498
H	-0.857	3.216	22.07
H	-1.669	1.885	21.712
H	-0.432	1.819	22.725
H	-3.347	0.992	23.283
H	-3.298	1.451	24.817
H	-2.066	0.651	24.182

H	1.802	1.291	26.821
H	2.267	1.385	28.349
H	2.367	2.67	27.402
H	-0.424	0.456	27.503
H	-1.293	1.273	28.571
H	0.038	0.513	29.034
H	0.073	4.656	34.001
H	1.654	4.421	34.097
H	0.664	3.586	35.036
H	-0.656	1.81	34.234
H	-0.652	1.348	32.703
H	-1.387	2.705	33.128
H	1.839	1.556	34.113
H	2.687	2.465	33.106
H	1.799	1.26	32.542
H	1.071	7.16	30.606

$|g_{\text{lum}}| \propto (|\mathbf{m}|/|\boldsymbol{\mu}|)\cdot\cos\theta_{\mu,\text{m}}$[S6]

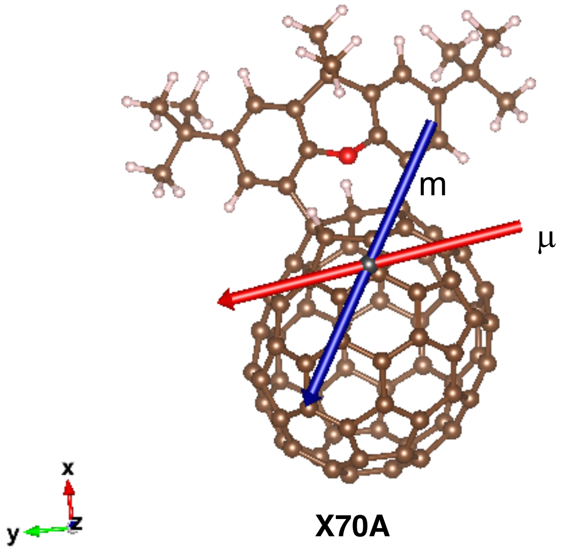

X70A

$|\boldsymbol{\mu}| = 2.52 \times 10^{-19}$ esu cm, $|\boldsymbol{m}| = 1.64 \times 10^{-20}$ erg G^{-1}, $\theta_{\mu,\text{m}} = 128°$, $\lambda = 611$ nm

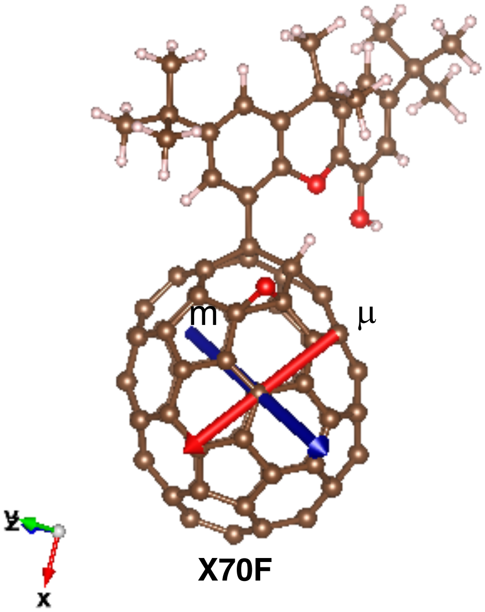

X70F

$|\boldsymbol{\mu}| = 6.77 \times 10^{-19}$ esu cm, $|\boldsymbol{m}| = 6.82 \times 10^{-21}$ erg G^{-1}, $\theta_{\mu,\text{m}} = 92°$, $\lambda = 607$ nm

Supplementary Fig. 28. Transition dipole moments of **X70A** and **X70F**. The electric transition dipole moment vector is shown in red, and the magnetic transition dipole moment vector is shown in blue.

References

[S1] Aikawa, K. & Nagata, T. Synthesis of a dinucleating ligand xanthene-bis(tris(2-pyridylmethyl)-amine) and its manganese complex. *Inorg. Chim. Acta* **306**, 223-226 (2000).

[S2] Nambo, M., Noyori, R. & Itami, K. Rh-Catalyzed arylation and alkenylation of C_{60} using organoboron compounds. *J. Am. Chem. Soc*. **129**, 8080-8081 (2007).

[S3] Gaussian 16, Revision C.01, Frisch, M.J., Trucks, G.W., Schlegel, H.B., Scuseria, G.E., Robb, M.A., Cheeseman, J.R., Scalmani, G., Barone, V., Petersson, G.A., Nakatsuji, H., Li, X., Caricato, M., Marenich, A.V., Bloino, J., Janesko, B.G., Gomperts, R., Mennucci, B., Hratchian, H.P., Ortiz, J.V., Izmaylov, A.F., Sonnenberg, J.L., Williams-Young, D., Ding, F., Lipparini, F., Egidi, F., Goings, J., Peng, B., Petrone, A., Henderson, T., Ranasinghe, D., Zakrzewski, V.G., Gao, J., Rega, N., Zheng, G., Liang, W., Hada, M., Ehara, M., Toyota, K., Fukuda, R., Hasegawa, J., Ishida, M., Nakajima, T., Honda, Y., Kitao, O., Nakai, H., Vreven, T., Throssell, K., Montgomery, J.A., Jr., Peralta, J.E., Ogliaro, F., Bearpark, M.J., Heyd, J.J., Brothers, E.N., Kudin, K.N., Staroverov, V.N., Keith, T.A., Kobayashi, R., Normand, J., Raghavachari, K., Rendell, A.P., Burant, J.C., Iyengar, S.S., Tomasi, J., Cossi, M., Millam, J.M., Klene, M., Adamo, C., Cammi, R., Ochterski, J.W., Martin, R.L., Morokuma, K., Farkas, O., Foresman, J.B. & Fox, D.J. Gaussian, Inc., Wallingford CT, 2016.

[S4] Yang, G., Si, Y. & Su, Z. Theoretical Study on the Chiroptical Optical Properties of Chiral Fullerene C_{60} Derivative, *J. Phys. Chem. A* **115**, 13356-13363 (2011).

[S5] a) Bruhn, T., Schaumlöffel, A., Hemberger, Y. & Pescitelli, G. SpecDis, Version 1.70.1, Berlin, Germany, https://specdis-software.jimdo.com (2017) b) Bruhn, T., Schaumlöffel, A., Hemberger, Y. & Bringmann, G. SpecDis: Quantifying the Comparison of Calculated and Experimental Electronic Circular Dichroism Spectra. *Chirality* **25**, 243-249 (2013). c) Bruhn, T. & Pescitelli, G. Good Computational Practice in the Assignment of Absolute Configurations by TDDFT Calculations of ECD Spectra. *Chirality* **28**, 466-474 (2016).

[S6] Sato, S., Yoshii, A., Takahashi, S., Furumi, S., Takeuchi, M. & Isobe, H. Chiral intertwined spirals and magnetic transition dipole moments dictated by cylinder helicity. *Proc. Nat. Acad. Sci*. **114**, 13097-13101 (2017).
